# Identification and characterization of a mosquito-specific eggshell organizing factor in *Aedes aegypti* mosquitoes

**DOI:** 10.1371/journal.pbio.3000068

**Published:** 2019-01-08

**Authors:** Jun Isoe, Lauren E. Koch, Yurika E. Isoe, Alberto A. Rascón, Heidi E. Brown, Brooke B. Massani, Roger L. Miesfeld

**Affiliations:** 1 Department of Chemistry and Biochemistry, The University of Arizona, Tucson, Arizona, United States of America; 2 Department of Chemistry, San José State University, San José, California, United States of America; 3 Mel and Enid Zuckerman College of Public Health, University of Arizona, Tucson, Arizona, Unites States of America; The Rockefeller University, UNITED STATES

## Abstract

Mosquito-borne diseases are responsible for several million human deaths annually around the world. One approach to controlling mosquito populations is to disrupt molecular processes or antagonize novel metabolic targets required for the production of viable eggs. To this end, we focused our efforts on identifying proteins required for completion of embryonic development that are mosquito selective and represent potential targets for vector control. We performed bioinformatic analyses to identify putative protein-coding sequences that are specific to mosquito genomes. Systematic RNA interference (RNAi) screening of 40 mosquito-specific genes was performed by injecting double-stranded RNA (dsRNA) into female *Aedes aegypti* mosquitoes. This experimental approach led to the identification of eggshell organizing factor 1 (EOF1, AAEL012336), which plays an essential role in the formation and melanization of the eggshell. Eggs deposited by EOF1-deficient mosquitoes have nonmelanized fragile eggshells, and all embryos are nonviable. Scanning electron microscopy (SEM) analysis identified that exochorionic eggshell structures are strongly affected in EOF1-deficient mosquitoes. EOF1 is a potential novel target, to our knowledge, for exploring the identification and development of mosquito-selective and biosafe small-molecule inhibitors.

## Abstract 2

An RNAi functional screen of 40 Aedes aegypti genes specific to the mosquito lineage helped to identify EOF1, a protein that plays an essential role in mosquito eggshell formation and melanization.

## Author summary

Mosquito-borne pathogens infect millions of people worldwide, and the rise in insecticide resistance is exacerbating this problem. A new generation of environmentally safe insecticides will be essential to control insecticide-resistant mosquitoes. One potential route to such novel insecticide targets is the identification of proteins specifically needed for mosquito reproduction. Female mosquitoes feed on blood to produce eggs, which are covered with an eggshell; using RNA interference screening of mosquito-specific genes in *Aedes aegypti* (the mosquito that transmits yellow fever), we identified the eggshell organizing factor 1 (EOF1) protein that plays an essential role in eggshell melanization and embryonic development. Nearly 100% of eggs laid by EOF1-deficient females had a defective eggshell and were not viable. Bleach assays on eggs further confirmed that mosquito-specific EOF1 is required for embryonic development in *A*. *aegypti*. Additional experiments revealed that EOF1 also plays an essential role in eggshell formation in *Aedes albopictus* (the tiger mosquito, a carrier of Zika virus and dengue fever). We hypothesize that EOF1 has evolved within the Culicidae family to effect eggshell formation and therefore maximize egg survival. The results provide new insights, to our knowledge, into mosquito egg maturation and eggshell synthesis and could lead to key advances in the field of mosquito vector control.

## Introduction

Developing new strategies for vector control is becoming critical because worldwide cases of *Aedes aegypti*-transmitted dengue and Zika virus infections have risen dramatically in the last decade [1–3]. Researchers have been investigating metabolic regulation of blood meal metabolism in *A*. *aegypti* mosquitoes as a strategy for identifying novel protein targets that could be exploited for vector control [4–14]. The approach has been focused on biochemical processes that are likely to be required for completion of the gonotrophic cycle in blood-fed mosquitoes, based on what is known about mosquito biology and metabolic regulation in other organisms. Specific genes in these chosen pathways were then systematically knocked down by microinjection of double-stranded RNA (dsRNA), and the resulting phenotypes were characterized in detail by molecular and biochemical approaches.

The insect eggshell is important as a protective layer for embryonic development. Follicle development and eggshell formation in the *A*. *aegypti* mosquito are tightly regulated in response to blood feeding [15–20]. Once female mosquitoes acquire blood, follicle development is initiated via accumulation of vitellogenin yolk proteins. Mosquitoes contain approximately 100 ovarioles per ovary, which are composed of primary and secondary follicles and a germarium, and the ovarian follicles develop synchronously throughout oogenesis (S1 Fig). A single layer of follicular epithelial cells surrounding the oocyte is mainly responsible for secreting a majority of eggshell structural components. The mosquito eggshell is made from different types of proteins ranging from structural proteins, enzymes, odorant binding proteins, and uncharacterized proteins of unknown function. *A*. *aegypti* eggshell melanization proteins were identified more than 20 years ago [21], and several key eggshell enzymes have been well characterized [22–28]. Moreover, proteomic studies have been performed on purified mosquito eggshells to identify most of the abundant protein components [29,30]. However, these descriptive studies have not identified essential eggshell proteins that are required for successful embryonic development and larvae viability.

Genomic sequences of *Drosophila melanogaster* [31], *Anopheles gambiae* [32], *A*. *aegypti* [33], and *Culex quinquefasciatus* [34] have been completed. Not surprisingly, many predicted putative proteins identified in the genome of mosquitoes are homologous to proteins of known function studied in other organisms. Proteins that are conserved in a wide variety of organisms are not ideal target molecules as vector control agents because of deleterious effects on nontarget organisms such as vertebrates, pollinating agricultural insects, and beneficial predators. We reasoned that if small-molecule inhibitors could be designed to exclusively target mosquito-lineage–specific proteins, they could be used as biosafe vector control agents. The objective of this study is to identify mosquito-specific proteins that are essential for supporting embryonic development in *A*. *aegypti* mosquitoes by RNA interference (RNAi) screening. Our findings indicate that eggshell organizing factor 1 (EOF1, AAEL012336) is necessarily required for mosquito eggshell formation and melanization.

## Results

### Identification of EOF1 as an essential protein for viable embryos

**Fig 1:**
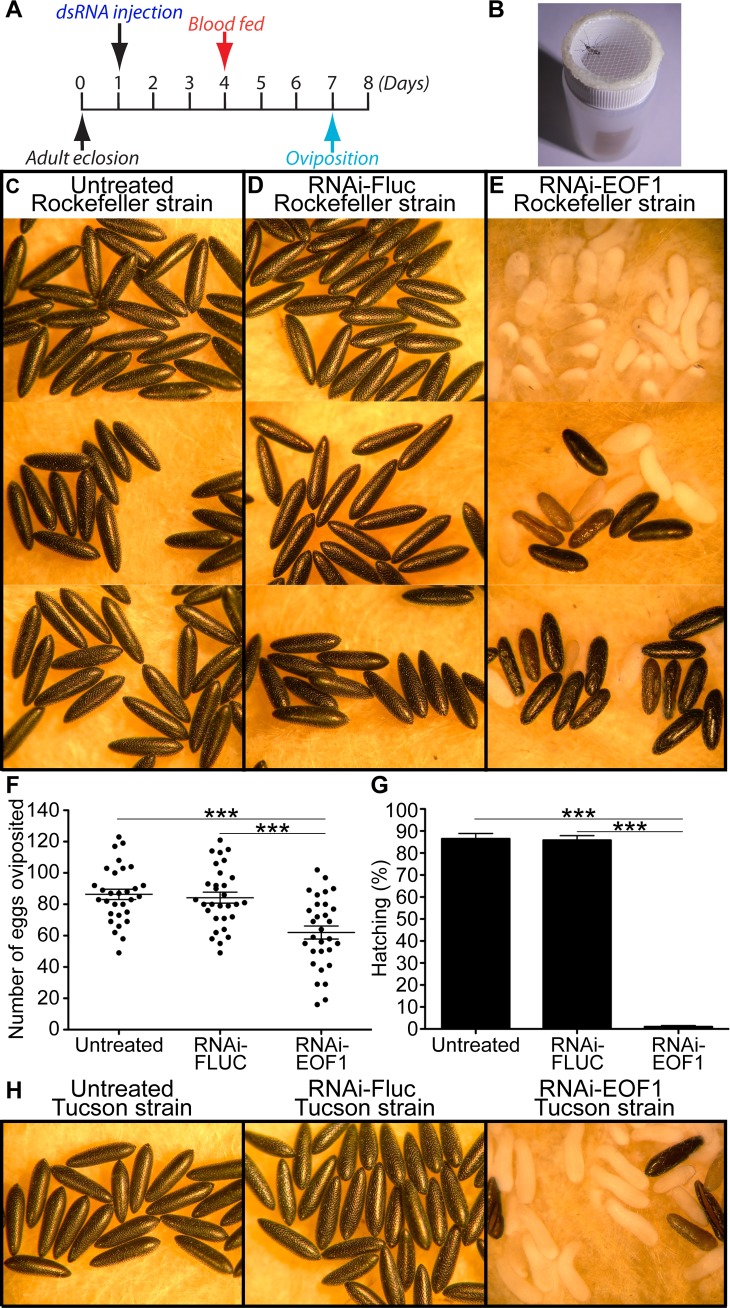
Egg phenotypes associated with EOF1 gene suppression by RNAi in *A*. *aegypti* mosquitoes. (A) Schematic diagram of experimental time course for dsRNA microinjection, blood feeding, and oviposition. (B) The photo image shows an oviposition experimental setup. Gravid female mosquitoes were placed in individual 15 ml scintillation vials for egg laying. Representative eggs are shown from three untreated mosquitoes (C), mosquitoes injected with dsRNA-Fluc (D), and dsRNA-EOF1–injected mosquitoes (E). Knockdown of EOF1 in female mosquitoes resulted in the production of nonmelanized abnormal eggs. (F) The effect of RNAi-EOF1 or RNAi-Fluc control on *A*. *aegypti* fecundity was examined by counting the number of eggs laid by each individual female. Note that 25% of fully blood-fed RNAi-EOF1 females did not produce mature follicles. Each dot represents the number of eggs oviposited by an individual mosquito (*N* = 30). The mean ± SE are shown as horizontal lines, and statistical significance is represented by stars above each column (unpaired Student's *t* test; ***p < 0.001). (G) Viability of these eggs was determined. A significant reduction in the percentage of egg hatching was observed in RNAi-EOF1 mosquitoes. Each bar corresponds to egg viability from 15 individual mosquitoes from three groups. (*H*) The influence of RNAi-EOF1 on egg phenotypes in *A*. *aegypti* Tucson strain was similar to those from the Rockefeller strain. Underlying data can be found in S1 Data. dsRNA, double-stranded RNA; EOF1, eggshell organizing factor 1; Fluc, firefly luciferase; RNAi, RNA interference; SE, standard error.

We performed data mining and bioinformatic analysis using the GenBank database to identify putative protein-coding sequences that are only present in the genomes of *Aedes*, *Culex*, and *Anopheles* mosquitoes using a cutoff for expected value threshold of 1 × 10^(−15). Importantly, the mosquito-lineage–specific genes we identified (S1 Table) were found to be completely absent in evolutionarily closely related organisms, such as phantom midges, true midges, the crane fly, and sandflies within the suborder Nematocera, and thus these genes are not present in other known animals, plants, fungi, and bacteria species. In order to focus on genes that are expressed and likely to encode proteins that could potentially serve as vector control targets, we excluded genes without corresponding messenger RNA (mRNA) in *A*. *aegypti* expressed sequence tags (ESTs) or expressed orthologs in the *Aedes albopictus* transcriptome shotgun assembly (TSA) database. We also excluded mosquito-lineage–specific genes that appear to be members of a multigene family because RNAi knockdown phenotypes may not be immediately obvious because of possible functional redundancy with other gene family members. This highly selected subset of hypothetical mosquito-lineage–specific proteins may have therefore evolved independently and advantageously within the family Culicidae. Systematic RNAi screening of mosquito-specific genes was performed by directly microinjecting the corresponding dsRNA into female *A*. *aegypti* mosquitoes 3 days prior to blood feeding (Fig 1A), and the blood-fed female mosquitoes were individually analyzed for their egg phenotypes, fecundity, and viability (Fig 1B). 40 mosquito-specific genes were screened (S1 Table), and utilizing this experimental approach led to the identification of EOF1, which, upon RNAi knockdown, plays an essential role in the strength and structural integrity of the forming eggshell, as well as its melanization. We hypothesize that EOF1 has evolved within the family Culicidae to affect eggshell formation and melanization and therefore maximize egg survival. We did not observe any defective eggshell in mosquitoes microinjected with dsRNA against 39 other putative genes in *A*. *aegypti* mosquitoes, resulting in the production of viable eggs. We also did not observe any significant mortality in response to RNAi against these genes. Thus, we chose to focus on EOF1 in subsequent analyses.

EOF1 sequences found in *Aedes*, *Culex*, and *Anopheles* mosquito species contain an F-box functional motif, and members of the F-box protein family are in general characterized by an approximately 50 amino acid F-box motif that interacts with a highly conserved SKP1 protein in the E3 ubiquitin ligase SCF complex [35], suggesting that EOF1 may function to regulate intracellular protein turnover. A further RNAi study on proteins that participate in the SCF complex was not pursued since these proteins that participate in the complex are relatively highly conserved across taxa, and therefore these proteins may not be ideal target proteins to further characterize in mosquitoes. In addition, RNAi knockdown against proteins in the SCF complex may have effects on other proteins that contain the F-box motif. Recent proteomic analysis has identified over 100 mosquito eggshell proteins [29–30], and some of these proteins identified are enzymes that may be involved in catalyzing eggshell melanization and cross-linking reactions [22–28]. However, EOF1 was not previously identified in these mosquito eggshell proteomic studies, indicating that EOF1 may be an upstream regulatory factor of eggshell proteins. As shown in Fig 1, injection of dsRNA-EOF1 had a significant adverse impact on eggshell formation and egg viability. Single-mosquito analysis showed that phenotypes associated with RNAi-EOF1 range from totally nonmelanized and collapsed to truncated and melanized eggs, while untreated and RNAi-firefly luciferase (Fluc) control mosquitoes laid eggs that exhibit uniformly elongated and melanized patterns (Fig 1C–1E). Approximately 60% of eggs laid by EOF1-deficient mosquitoes did not show any melanization (Fig 1C), while 30% of them had mixed melanization levels ranging from nonmelanized and partially melanized to completely melanized eggshells (Fig 1C). On the other hand, 10% of eggs had completely or partially melanized eggshells (Fig 1C). Overall, nearly 100% of eggs from any melanization levels did not reach the larval stage. Single-mosquito analysis also showed that fecundity and viability from eggs of RNAi-EOF1 females were strongly affected by reduced EOF1 function through RNAi (Fig 1F and 1G). Bleaching experiments on eggs further confirmed that mosquito-specific EOF1 is required for embryonic development in *A*. *aegypti* mosquitoes. Under a light microscope, we observed eggs throughout the 2 h eggshell dechorionation experiment period. In the majority of eggs laid by EOF1-deficient mosquitoes, embryos failed to complete embryogenesis and reach the first larval instar (S2 Fig). It has been shown that when a mosquito embryo advances to form a serosal cuticle within the eggshell, bleach treatment was found to only remove the eggshell, but it cannot digest the serosal cuticle, leaving an intact embryo [36]. Thus, if a serosal cuticle has been formed during embryogenesis, the embryo or developed larva should be resistant to bleach treatment. A recently colonized *A*. *aegypti* Tucson strain from wild populations [37] also exhibited similar defective egg and embryo phenotypes associated with RNAi-EOF1 (Fig 1H). We analyzed 15 mosquitoes from each group, and the fecundity (mean ± standard error [SE]) of eggs was 33.3 ± 3.0, 26.4 ± 2.8, and 17.5 ± 1.8 (unpaired Student's *t* test; *p* < 0.01) for untreated, RNAi-Fluc, and RNAi-EOF1, respectively. The lower number of eggs laid by the Tucson strain compared to the Rockefeller strain of *A*. *aegypti* could likely be due to reduced blood ingestion. The viability of eggs (mean ± SE) was 88.4 ± 2.1, 87.0 ± 1.7, and 2.4 ± 0.9% (unpaired Student's *t* test; *p* < 0.001) for untreated, RNAi-Fluc, and RNAi-EOF1, respectively. Thus, EOF1 protein is essential for complete eggshell formation and embryonic development in *A*. *aegypti* mosquitoes.

**Fig 2:**
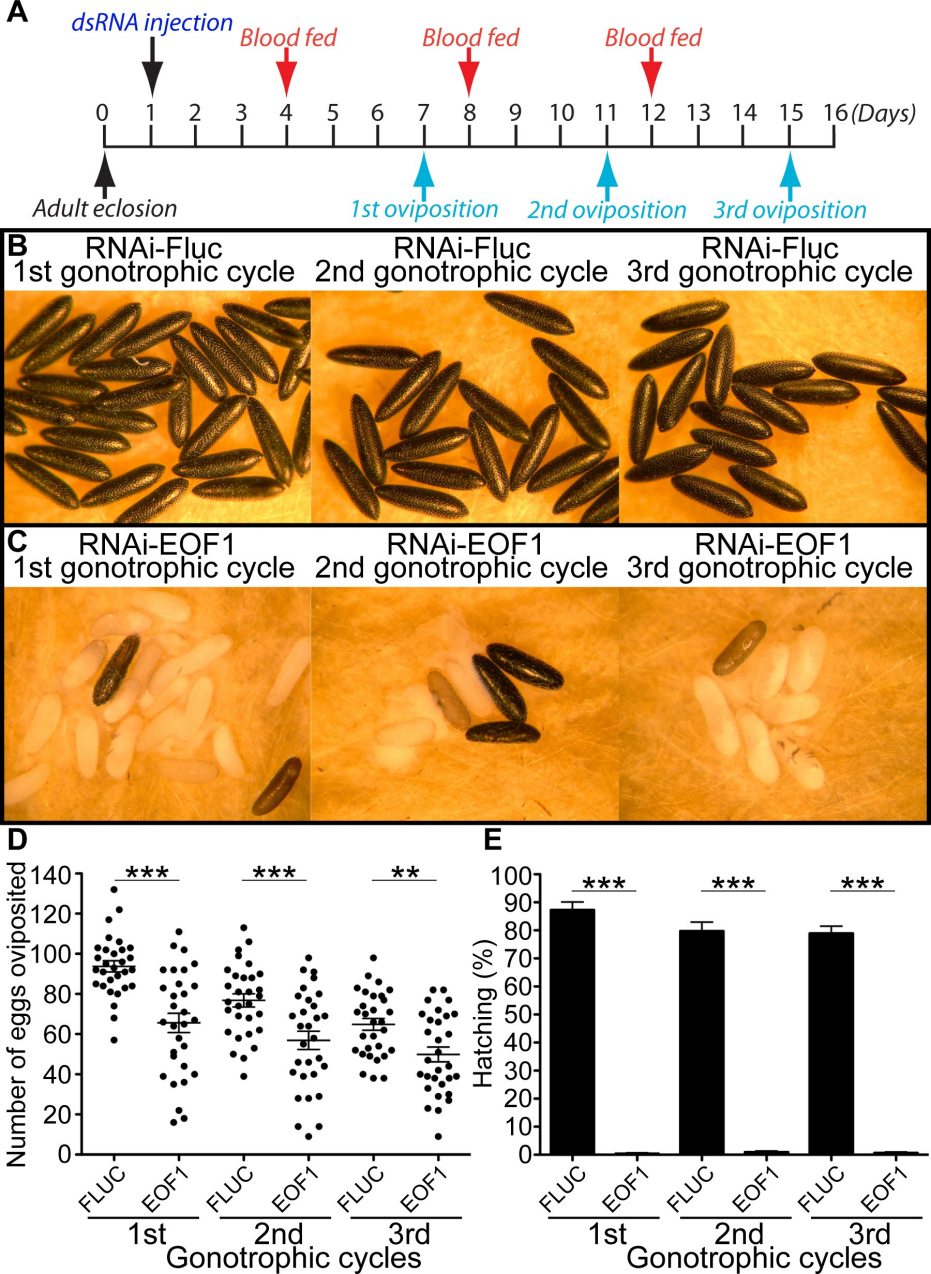
A single microinjection of dsRNA against EOF1 promotes the abnormal eggshell phenotypes in multiple gonotrophic cycles. (*A*) Schematic diagram of experimental time course for dsRNA microinjection, blood feeding, and oviposition in the first three gonotrophic cycles. Representative eggs are shown from mosquitoes microinjected with dsRNA-Fluc (B) and dsRNA-EOF1 (C). An effect of RNAi-EOF1 or RNAi-Fluc control on fecundity was studied during the first three gonotrophic cycles (D). Each dot represents the number of eggs oviposited by an individual mosquito (*N* = 30). The mean ± SE are shown as horizontal lines, and statistical significance is represented by stars above each column (unpaired Student's *t* test; ***p < 0.001; **p < 0.01). An effect of RNAi-EOF1 on viability was also examined during the first three gonotrophic cycles (E). A significant reduction in the percentage of egg hatching was observed in RNAi-EOF1 mosquitoes. Each bar corresponds to egg viability from 15 individual mosquitoes from both groups. Underlying data can be found in S1 Data. dsRNA, double-stranded RNA; EOF1, eggshell organizing factor 1; Fluc, firefly luciferase; RNAi, RNA interference; SE, standard error.

Anautogenous female mosquitoes can undergo multiple gonotrophic cycles by repeating blood feeding, vitellogenesis, and oviposition events. Because EOF1 plays an essential role in eggshell formation, we wondered how long the RNAi knockdown effect of EOF1 lasts from a single dsRNA microinjection. We examined the effect of EOF1 deficiency on eggs in three consecutive gonotrophic cycles in individual containers as designed in Fig 2A and Fig 1B. Eggshell melanization (Fig 2B and 2C), fecundity (Fig 2D*)*, and viability (Fig 2E) phenotypes are profoundly altered in EOF1-deficient mosquitoes during the first three gonotrophic cycles. Therefore, our data demonstrate that the RNAi-EOF1 effect from a single dsRNA injection remains substantial for the second and even the third gonotrophic cycles. Furthermore, we found that the timing of dsRNA microinjection is important. The dsRNA has to be microinjected a few days prior to blood feeding in both the first and second gonotrophic cycles in order to induce RNAi-mediated EOF1 depletion and produce defective egg phenotypes (S3 Fig).

### EOF1 expression pattern

**Fig 3:**
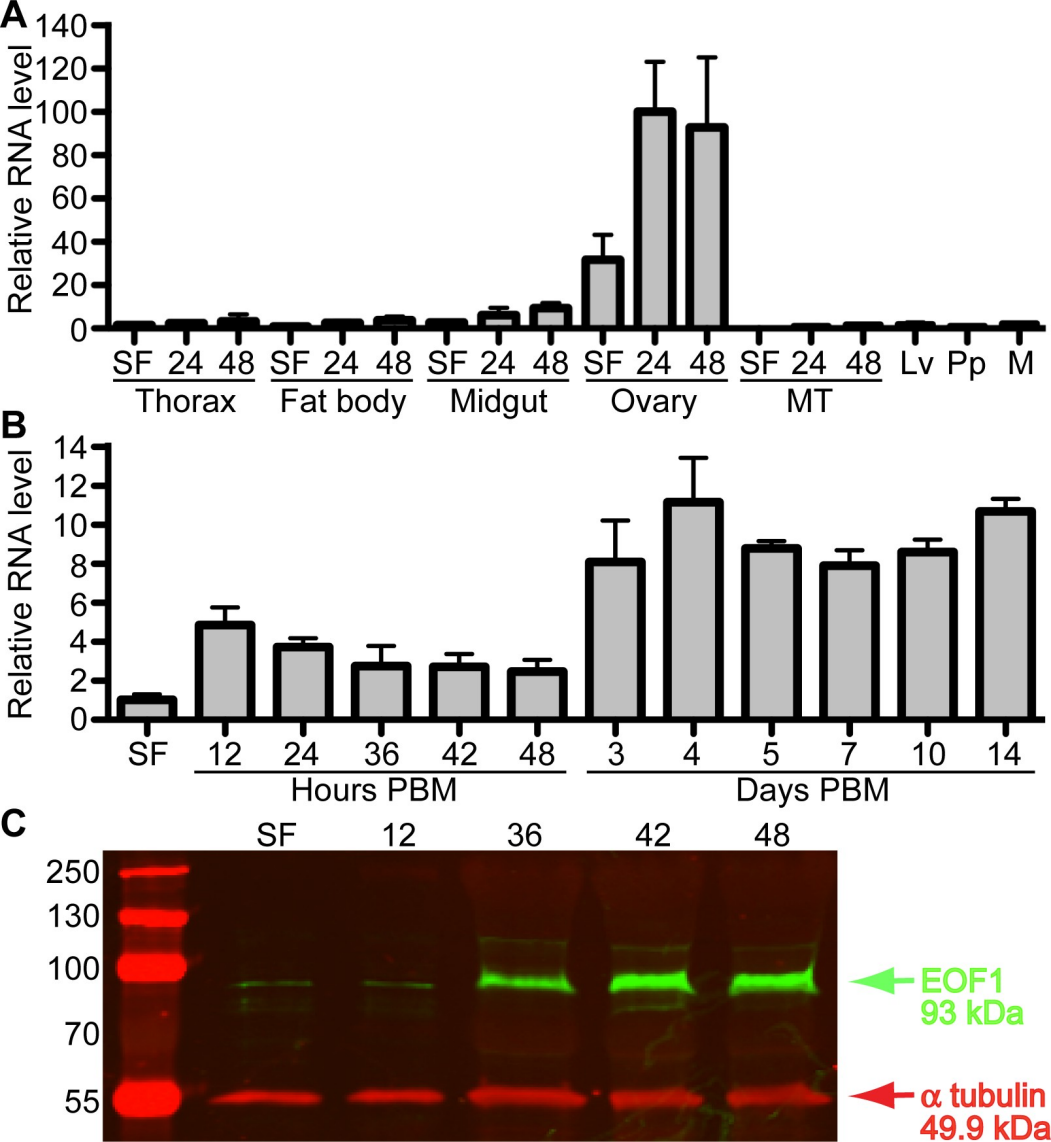
Spatial distribution and expression level of EOF1 during the first gonotrophic cycle in *A*. *aegypti*. (A) Tissue-specific and developmental expression pattern of EOF1 during the first gonotrophic cycle of *A*. *aegypti* mosquitoes was determined. EOF1 gene expression was analyzed by qPCR using cDNAs prepared from various tissues. Tissues include thorax, fat body, midgut, ovary, and MTs in SF only and 24 and 48 h PBM, as well as Lv, Pp, and M. The pattern demonstrates the ovary-specific EOF1 expression in *A*. *aegypti* mosquitoes. The EOF1 expression levels were normalized to S7 ribosomal protein transcript levels in the same cDNA samples. Data were collected from three different mosquito cohorts. EOF1 expression in fat body at SF was set to 1.0. (B) Detailed EOF1 gene expression in ovaries and follicles were analyzed by qPCR using cDNAs from mosquito ovaries or follicles. Samples from SF to 42 h PBM include entire ovaries, whereas those from 48 h to 14 days PBM include only follicles (primary and secondary follicle cells and germarium) isolated from ovaries to exclude nonfollicle cells in the oviducts and ovarioles. EOF1 expression in ovaries at SF was set to 1.0. (C) EOF1 protein expression pattern in ovaries were determined by western blot analysis using an EOF1-specific polyclonal antibody. Each lane contains 0.3 ovary equivalent of protein extracts. α-tubulin was used as an internal control. qPCR and western blot experiments were performed in triplicate. Underlying data can be found in S1 Data. cDNA, complementary DNA; EOF1, eggshell organizing factor 1; Lv, larvae; M, adult male; MT, Malphigian tubule; PBM, post-blood meal; Pp, pupae; qPCR, quantitative real-time PCR; SF, sugar fed.

Since little is known about this mosquito-specific EOF1 gene except for the phenotypes associated with RNAi, we determined the expression pattern of EOF1 at the mRNA level in untreated *A*. *aegypti* by quantitative real-time PCR (qPCR). Five tissues including thorax, fat body, midgut, ovaries, and Malpighian tubules were dissected from sugar-fed female mosquitoes at 3 days posteclosion and blood-fed female mosquitoes at 24 and 48 h post-blood meal (PBM). We also examined the mRNA expression in whole bodies of mixed-sex samples of fourth-instar larvae and pupae and adult male mosquitoes. EOF1 is predominantly expressed in ovaries, and the expression is up-regulated in the ovaries by blood feeding (Fig 3A). qPCR results also indicate that mRNA encoding EOF1 is not strongly detected from larvae, pupae, and adult male mosquitoes. We then examined the pattern of EOF1 expression during the first gonotrophic cycle in detail. Ovaries were isolated at various time points PBM. In ovary samples after 36 h PBM, the primary follicles were carefully isolated from ovaries to exclude nonfollicle ovarian cell types such as muscles and trachea. qPCR data show that EOF1 mRNA expression is up-regulated in response to blood feeding, and the levels remain high even at 14 days PBM (Fig 3B). Since follicular epithelial cells and nurse cells in the primary follicles undergo apoptosis by around 72 h PBM (S4 Fig), mRNAs encoding EOF1 may likely originate from the unfertilized mature oocytes. EOF1 mRNA distribution in primary follicles was further determined using whole-mount fluorescent in situ hybridization (FISH). FISH analysis shows that while three vitelline envelope genes [38] were exclusively expressed in the follicular epithelial cells, EOF1 mRNA transcripts are present in oocyte and nurse cells of primary follicles and weakly expressed in the secondary follicle and germarium (S5 Fig). Western blot analysis showed that EOF1 expression is induced in ovaries in response to blood feeding (Fig 3C). RNAi knockdown level of EOF1 mRNA and protein was confirmed by qPCR and western blot, respectively (S6 Fig).

### Follicle development in EOF1-deficient females

**Fig 4:**
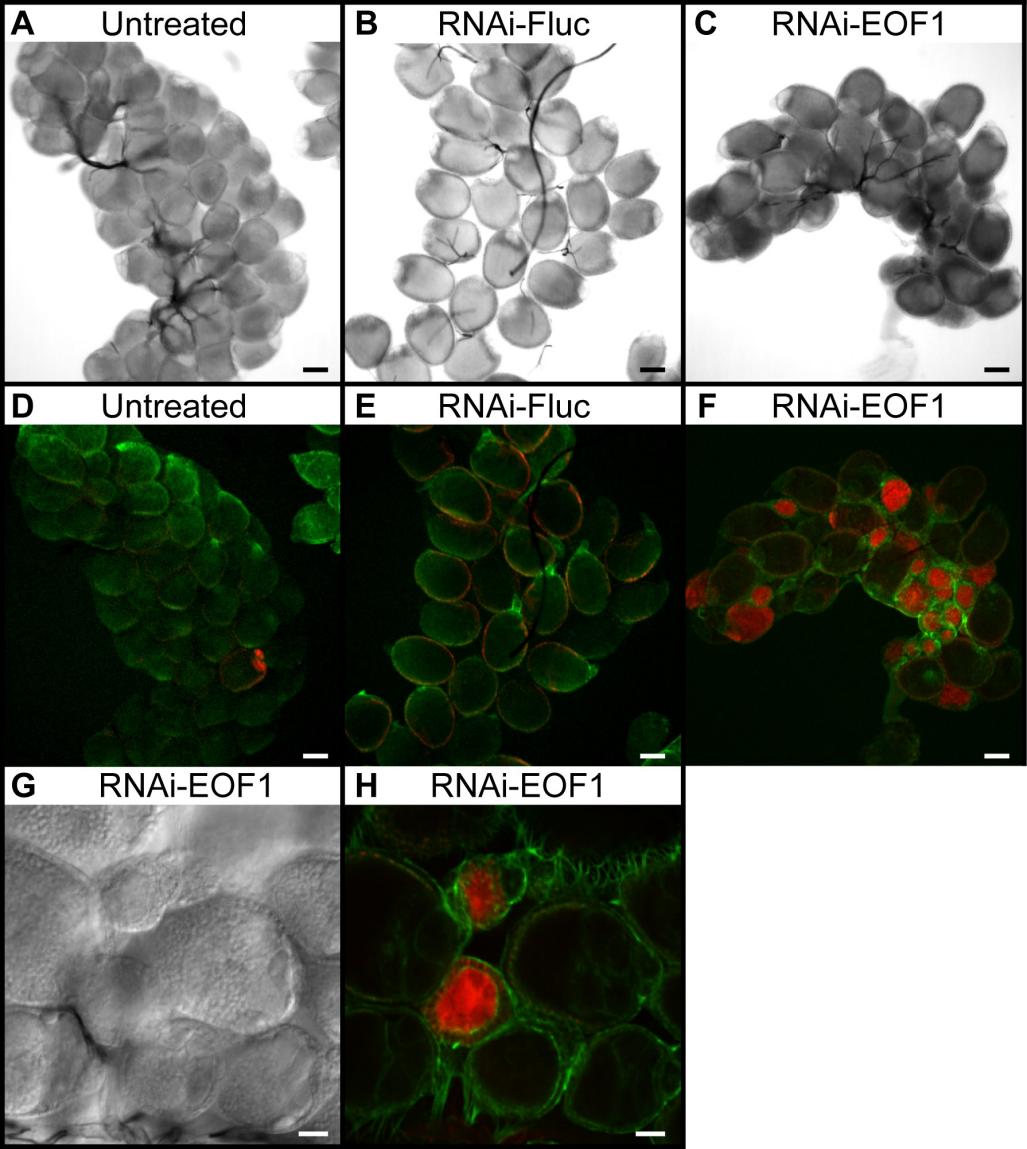
EOF1 gene knockdown by RNAi initiates a high degree of caspase-mediated apoptosis in ovarian follicles of *A*. *aegypti* mosquitoes. Light microscopic images of representative ovaries were taken from untreated (A), RNAi-Fluc (B), and RNAi-EOF1 (C). Mosquitoes were dissected at 36 h PBM, and dissected live ovaries were incubated with a caspase inhibitor (red), fixed with 4% paraformaldehyde, and stained with phalloidin (green). Confocal images of the corresponding ovaries were taken from untreated (D), RNAi-Fluc (E), and RNAi-EOF1 (F) mosquitoes. Scale bar for the images (A–F) is 100 μm. A high degree of caspase-mediated apoptosis was observed in the follicles of ovaries from RNAi-EOF1 females, possibly resulting in the lower fecundity as shown in Fig 1F. DIC (G) and confocal (H) images of ovaries from RNAi-EOF1 mosquitoes are taken at a higher magnification (scale bar: 50 μm). Caspase activity was more restricted to the oocytes, rather than in the follicular epithelial cells. Images (A–H) were collected with a spinning disk confocal microscope. Ovaries from 10–15 individual mosquitoes from each group were analyzed. DIC, differential interference contrast; EOF1, eggshell organizing factor 1; Fluc, firefly luciferase; PBM, post-blood meal; RNAi, RNA interference.

In *A*. *aegypti*, EOF1-deficient female mosquitoes had low fecundity (Fig 1F) and laid eggs that were defective in eggshell formation, leading to the embryonic lethal phenotype (Fig 1G). Similar reproductive phenotypes associated with RNAi-EOF1 were found in *A*. *albopictus* (S7 Fig). EOF1 is required for proper eggshell formation, fecundity, and viability. We hypothesized that primary follicles of EOF1-deficient mosquitoes undergo cell death, removing severely affected follicles within the ovaries. We examined ovarian follicle phenotypes associated with EOF1 gene suppression by RNAi in *A*. *aegypti* mosquitoes. Representative ovaries at 36 h PBM showed that RNAi-EOF1 ovaries contain follicles that undergo caspase-mediated apoptosis indicated by the increase in red-labeled caspase inhibitor, while these dying follicles were not observed in untreated or RNAi-Fluc control ovaries (Fig 4). Approximately 40% of primary follicles in RNAi-EOF1 ovaries showed caspase-mediated apoptosis. Differential interference contrast and confocal images at a higher magnification showed that the caspase activity was more concentrated in the oocytes than in the follicular epithelial cells from RNAi-EOF1 mosquitoes (Fig 4G and 4H).

**Fig 5:**
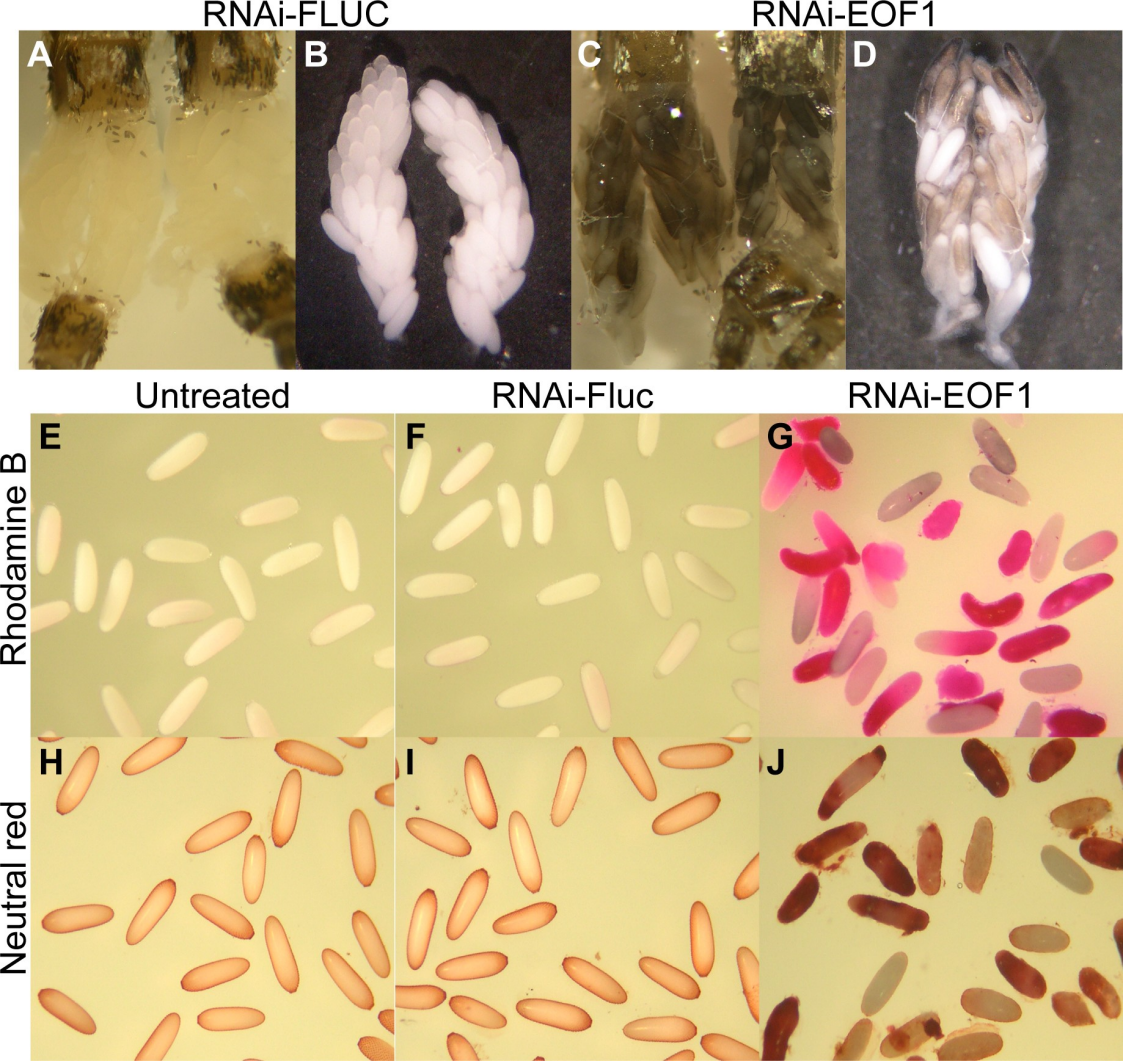
Ovarian follicle phenotypes associated with EOF1 gene suppression by RNAi in *A*. *aegypti* mosquitoes. Representative ovaries at 96 h PBM from mosquitoes injected with dsRNA-Fluc (A and B) and dsRNA-EOF1 (C and D). Ovaries were photographed immediately after dissection in 1× PBS. RNAi-Fluc control mosquitoes contain mature nonmelanized ovarian follicles, whereas EOF1-deficient mosquitoes contain aberrant prematurely melanized follicles. Rhodamine B and neutral red were used to stain mature primary follicles isolated from mosquito ovarian follicles isolated at 96 h PBM (E–J). The representative stained images of untreated (E and H), RNAi-Fluc (F and I), and RNAi-EOF1 follicles (G and J). The follicle permeability assays show a strongly increased rhodamine B and neutral red uptake by RNAi-EOF1 follicles, whereas the follicles from untreated and RNAi-Fluc control mosquitoes were not stained. A loss of EOF1 mediated by RNAi may be responsible for defective eggshell formation. dsRNA, double-stranded RNA; EOF1, eggshell organizing factor 1; Fluc, firefly luciferase; PBM, post-blood meal; RNAi, RNA interference.

Mature ovaries were dissected from the abdomen of dsRNA-injected mosquitoes and photographed at 96 h PBM (Fig 5A–5D). While not all RNAi-Fluc control mature follicles in ovaries have initiated melanization, we frequently observed that some follicles isolated from RNAi-EOF1 are already partially melanized in the ovaries. The partially melanized phenotype in EOF1-deficient ovaries is accompanied by a loss of structural integrity, and thus we hypothesized that decreased chorionic osmotic control results in this alteration of egg shape. To determine whether the water permeability of the mosquito eggshells was affected in response to EOF1 knockdown, we employed two chemical markers, rhodamine B and neutral red, to stain ovarian follicles. Significant differences in the permeability of both markers in ovaries were observed (Fig 5E–5J). While the follicles from both untreated and RNAi-Fluc mosquitoes were only slightly stained, the majority of follicles from RNAi-EOF1 mosquitoes were strongly stained with the markers. Since follicular epithelial cells have been already shed around 72 h PBM (S4 Fig), there is a single oocyte present in each follicle at this developmental stage (96 h PBM). The reduction of EOF1 expression in female mosquitoes resulted in defective eggshells, leading to increased permeability of water into oocytes (Fig 5G and 5J) and altered follicular shape.

### Ultrastructure analysis of mosquito eggs

**Fig 6:**
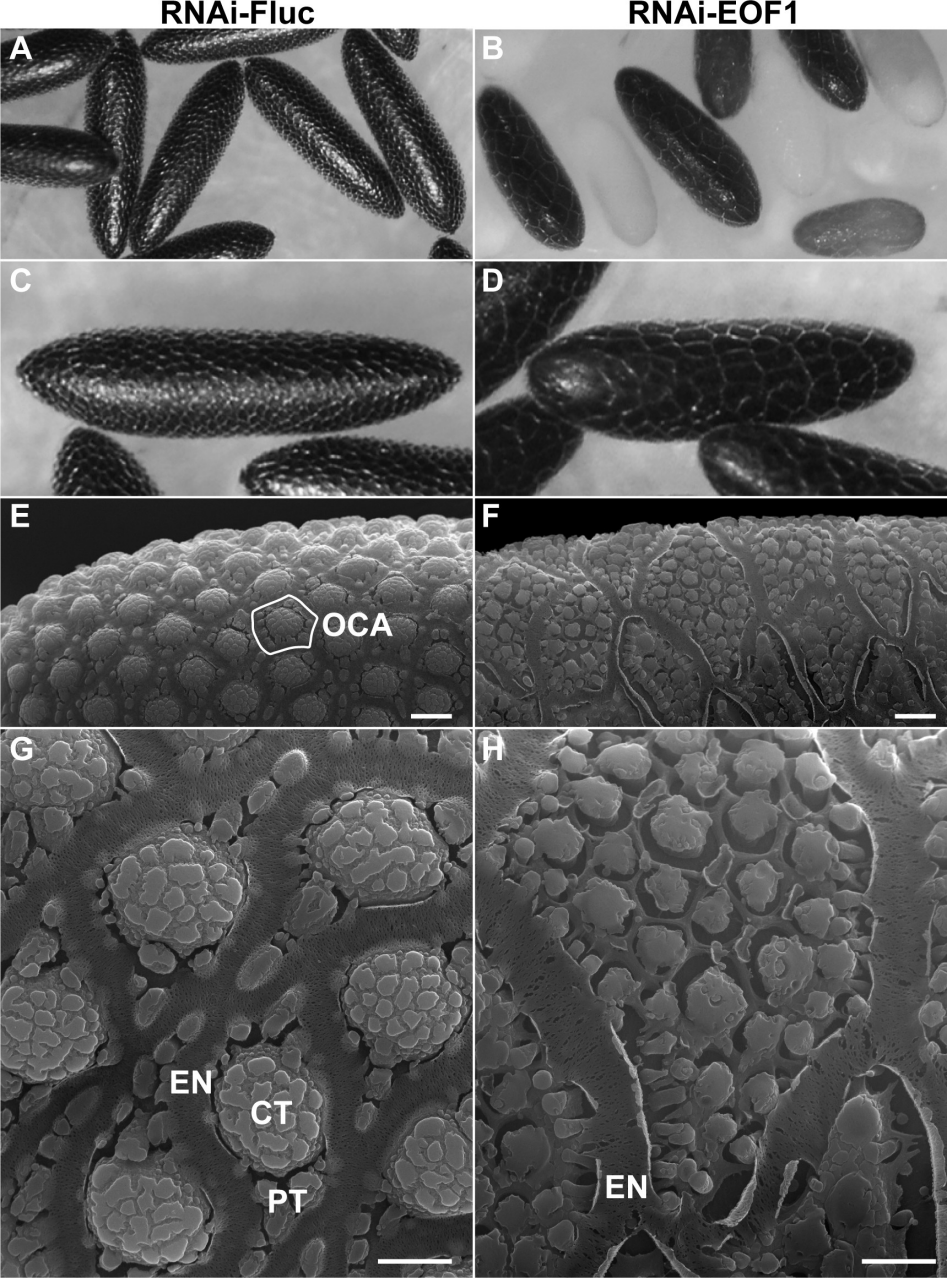
EOF1 regulates eggshell ultrastructure in *A*. *aegypti* mosquitoes. Representative light microscope images of *A*. *aegypti* eggs are shown for RNAi-Fluc (A and C) and RNAi-EOF1 (B and D). Representative scanning electron micrograph images of eggs from RNAi-Fluc (E and G) and RNAi-EOF1 (F and H) were taken utilizing an FEI Inspect-S electron scanning microscope at the Keck Imaging Center at the University of Arizona. The magnification is 2,000× (E and F; scale bars = 10 μm) and 8,000× (G and H; scale bars = 5 μm). Mosquitoes were injected with dsRNA against Fluc and EOF1 prior to feeding as shown in Fig 1A. The primary follicles were dissected in 1× PBS at 96 h PBM, immediately fixed with 2.5% glutaraldehyde at 4°C, postfixed at room temperature for 1 h with 1% osmium tetroxide, dried with HMDS, and metalized with gold. CT, central tubercle; dsRNA, double-stranded RNA; EN, exochorionic network; EOF1, eggshell organizing factor 1; Fluc, firefly luciferase; HMDS, hexamethyldisilazane; OCA, outer chorionic area; PBM, post-blood meal; PT, peripheral tubercle; RNAi, RNA interference.

EOF1-deficient mosquitoes oviposited eggs with different degrees of eggshell melanization phenotypes that include nonmelanized, partially melanized, and melanized eggs (Figs 1E and 2C). Since nearly 100% of eggs oviposited from RNAi-EOF1 females did not undergo complete embryogenesis (Figs 1G and 2E), we hypothesized that the defective eggshell might be the primary cause of embryonic death. Light microscopy images of eggs from *A*. *aegypti* RNAi-Fluc and RNAi-EOF1 mosquitoes revealed that EOF1 may be involved in the specification of the outer chorionic area (OCA) surrounded by the porous nature of the exochorionic network (EN) (Fig 6A–6D*)*. Next, we examined the effect of RNAi-EOF1 on the ultrastructure of eggs in detail by scanning electron microscopy (SEM). We observed a very similar *A*. *aegypti* eggshell ultrastructure (Fig 6E and 6G) to other SEM studies [39,40]. An exochorion outermost layer of the eggshell is characterized by the presence of a single protruding central tubercle (CT) and several minute peripheral tubercles (PTs) in the OCA (Fig 6G). However, SEM images showed that the OCA in RNAi-EOF1 eggs is about 6 times larger than eggs of control mosquitoes (Fig 6E–6H), suggesting that EOF1 may be involved in specifying the size of the OCA. A majority of eggshell proteins are likely secreted into the perivitelline space from follicular epithelial cells during follicle development in response to blood feeding. However, it is not well known whether the size of the OCA is strictly determined by surrounding follicular epithelial cells. We also observed that each OCA contains multiple miniaturized CT-like structures also surrounded by EN-like structures instead of one predominant CT. The SDS-PAGE analysis demonstrated that the enriched eggshell protein extracts from EOF1-deficient females showed slightly different patterns from those from RNAi-Fluc controls (S8 Fig). These differences could account for aberrant exochorionic structure in RNAi-EOF1 eggs. Thus, EOF1 may act as an upstream factor to control eggshell surface patterning in *A*. *aegypti*.

## Discussion

Data mining was performed using all *A*. *aegypti* protein sequences available at the GenBank database in early 2015 to identify putative protein-coding sequences that are only present in the genomes of *Aedes*, *Culex*, and *Anopheles* mosquitoes. Through RNAi screening of putative mosquito-specific genes in *A*. *aegypti*, we identified EOF1 as an essential protein for eggshell formation and melanization. During this course of study, a whole-genome analysis in 37 dipteran species, including midges and sandflies, was performed [41]. We performed a TBLASTN search against Nematocera in the Whole-Genome-Shotgun contigs database. A global alignment between mosquito EOF1 and hypothetical proteins detected in culicoid and chaoborus midges shows only about 26%–30% identity, suggesting that it is very difficult to determine whether these highly diverged genes show orthologous relationships. The conserved amino acid residues are present in putative F-box functional domains of these proteins. Taken together, EOF1 may be uniquely evolved in mosquito lineages to play roles in eggshell formation.

We observed that EOF1-deficient mosquitoes lay eggs with different melanization levels, ranging from nonmelanized to completely melanized eggshells (Fig 1C). Different level of eggshell melanization in response to RNAi-EOF1 could be possible because of genetic variation. Alternatively, a differential dsRNA uptake by follicles within the ovary could explain the variation in the levels of eggshell melanization. A single layer of follicular epithelial cells surrounding the oocyte is mainly responsible for secreting a majority of eggshell structural components. Since eggshell components are directly secreted into the extracellular space between the oocyte and the surrounding follicular epithelial cells (S1 Fig), intimate communication between these cells within each ovariole may exist throughout follicle maturation, eventually leading to follicular epithelial cell shedding (S4 Fig), ovulation, and oviposition. In general, mature follicles from mosquitoes do not undergo premature melanization within the ovaries (Fig 5), and gravid females can hold their mature follicles for a long period of time under adverse environmental conditions and still lay viable eggs, which become melanized after oviposition. Thus, the timing of eggshell melanization may likely be tightly regulated and catalyzed by specific enzymes, and their synthesis, secretion, and activation may be critical for proper melanization and thus survival of embryos [22–28]. A possible explanation for aberrant partial melanization of follicles within the EOF1-deficient ovaries prior to an oviposition event is that a loss of EOF1 function may alter hemolymph permeability of the eggshell, affecting a delicate chemical balance within the oocytes, which in turn trigger other eggshell components to prematurely initiate eggshell melanization processes. In addition to *A*. *aegypti*, we also confirmed that EOF1 plays an essential role in eggshell formation in *A*. *albopictus* (S7 Fig). Given that *A*. *aegypti* embryogenesis completes by 3 days postoviposition [36], two lines of our experimental evidence suggest that EOF1 may be necessary for complete embryogenesis in *A*. *aegypti* mosquitoes. First, we frequently observed that a majority of eggs deposited by EOF1-deficient mosquitoes collapsed within 16 h, rupturing the oocyte plasma membrane and leaking intracellular contents, including yolk, likely because of incomplete eggshell formation. Second, our bleach assay demonstrates that nearly 100% of eggs from EOF1-deficient mosquitoes with any melanization levels did not reach the larval stage (S2 Fig). Thus, female mosquitoes without EOF1 produce inviable eggs likely because of incomplete embryogenesis. However, it remains to be determined which specific embryogenesis stage was affected in eggs deposited by EOF1-deficient mosquitoes. SDS-PAGE analysis shows that enriched eggshell proteins from EOF1-deficient eggs slightly differ from those from RNAi-Fluc controls (S8 Fig), and thus an identification of the downstream EOF1-dependent eggshell proteins may lead to a better understanding of molecular mechanisms for mosquito eggshell formation. Although it is beyond the scope of this study to screen and identify small molecules that specifically target mosquito EOF1, such molecules may have great promise for controlling the mosquito population. Eggshell proteins specifically affected by EOF1-deficient mosquitoes may also be ideal proteins if they exhibit a high degree of sequence divergence to other insect taxa.

Based on the presence of a conserved F-box motif in EOF1, one possibility is that EOF1 is required in the ubiquitin pathway for controlled degradation of one or more proteins that regulate proper timing of eggshell development. Dysregulation of stage-specific ordered events in RNAi-EOF1–injected mosquitoes could lead to collapse of the developmental program at all downstream control points. The finding that RNAi-EOF1 phenotypes are observed three gonotrophic cycles beyond the time of injection (Fig 2) indicates that the EOF1 protein may not be resynthesized at the onset of each gonotrophic cycle but rather establishes the eggshell development program when the reproductive phase is initiated in the female mosquito. The data in Fig 3 support this proposal in that EOF1 mRNA remains at elevated levels in the ovaries of blood-fed mosquitoes, even out to 14 days. Specifically, if EOF1 mRNA was resynthesized with each gonotrophic cycle, then one would expect EOF1 mRNA to be degraded at completion of the gonotrophic cycle in order to restart the process after the next blood feeding, and that does not appear to be the case. Another possibility is that RNAi effects are particularly long-lasting in ovary tissues and continue to abrogate EOF1 synthesis at each gonotrophic cycle. Additional studies are currently underway to distinguish between these alternative hypotheses. Results from these studies could lead to the development of mosquito control applications using novel biosafe mosquitocides that target mosquito-specific proteins required for embryonic development. It may also be possible to use clustered regularly interspaced short palindromic repeats (CRISPR) and CRISPR-associated protein 9 (CRISPR-Cas9) gene-drive genetic manipulation [42–46] for the same purpose. If successful, such approaches would eventually result in a decrease in the mosquito population and thus lower the transmission of mosquito-borne viral infections.

## Materials and methods

### Mosquitoes

Most of the experiments were carried out using *A*. *aegypti* mosquitoes (Rockefeller strain) and reared as previously described [5]. For comparison, *A*. *aegypti* mosquitoes (Tucson strain) were colonized from Tucson, Arizona [37]. *A*. *albopictus* (Gainesville strain, MRA-804) mosquitoes were obtained from CDC/MR4. Mosquitoes were maintained at 28°C, 72% relative humidity with a photoperiod of 16 h light and 8 h dark cycle in a CARON 6010 Insect Growth Chamber (Caron Products & Services, Marietta, OH, USA). The larvae were fed on a diet consisting of dog food, Tetramin fish food, and liver powder (10∶10:1 ratio). Male and female adults were maintained on 10% sucrose and kept together throughout all experiments until transferred to oviposition containers. Using an artificial glass feeder, female mosquitoes were allowed to feed on expired human blood donated by the American Red Cross (approved protocol #2010–017). Only fully engorged female mosquitoes were used.

### Identification of mosquito-specific putative genes

Data mining and bioinformatic analysis were carried out using all *A*. *aegypti* GI numbers available at the GenBank database. To identify putative protein-coding sequences that are only present in the genomes of *Aedes*, *Culex*, and *Anopheles* mosquitoes, we use NCBI BLASTP with default search methods and a cutoff for expected value threshold of 1e-15. Proteins selected were further subjected to NCBI TBLASTN search using *A*. *aegypti* ESTs or expressed orthologs in the *A*. *albopictus* TSA database to determine whether the genes are expressed. Mosquito-specific putative genes without corresponding mRNA in *A*. *aegypti* ESTs or *A*. *albopictus* TSA database were excluded as candidate genes for RNAi screening. We also excluded genes that appeared to be members of a multigene family because of possible functional redundancy with other gene family members. We also used a TBLASTN search from NCBI against Nematocera (taxid: 7148) in the Whole-Genome-Shotgun contigs database.

### dsRNA synthesis and microinjection

RNAi was carried out to knock down *A*. *aegypti* mosquito genes. Each gene-specific forward and reverse oligonucleotide primer was designed using a NetPrimer web-based primer analysis tool. A T7 RNA polymerase promoter sequence, TAATACGACTCACTATAGGGAGA, was added to the 5′ end of each primer (S1 Table). All primers were purchased from Eurofins Genomics (Louisville, KY, USA). PCR was performed using the Taq 2X Master Mix (NEB, Ipswich, MA, USA) with mosquito whole-body complementary DNA (cDNA) as a template, and the amplified PCR products were cloned into the pGEM-T easy vector (Promega Madison, WI, USA) for DNA sequence verification using an ABI 377 automated sequencer (Applied Biosystems, Foster City, CA, USA). dsRNA was synthesized by in vitro transcription using a HiScribe T7 Quick High Yield RNA Synthesis Kit (NEB). The purified dsRNA was resuspended with HPLC-grade water (Thermo Fisher Scientific, Waltham, MA, USA) at 7.3 μg/μL concentration. Cold-anesthetized female mosquitoes were injected with 2.0 μg dsRNA (276 nL) using a Nanoject II microinjector (Drummond Scientific Company, Broomall, PA). Injected mosquitoes were maintained on 10% sucrose throughout the experiments.

### Pattern of EOF1 gene expression by qPCR

Using TRIzol reagent (Thermo Fisher Scientific), total RNA was extracted from larvae, pupae, and male adults as well as five tissues including thorax, fat body, midgut, ovaries, and Malpighian tubules dissected from sugar-fed female mosquitoes at 3 days posteclosion and blood-fed female mosquitoes at 24 and 48 h PBM. First-strand cDNA was synthesized from pools of total RNA using an oligo-(dT)_20_ primer and reverse transcriptase. qPCR was carried out with the corresponding cDNA, EOF1, or ribosomal protein S7 gene-specific primers (S2 Table), PerfeCTa SYBR Green FastMix, and ROX (Quanta BioSciences, Gaithersburg, MD, USA) on the 7300 Real-Time PCR System (Applied Biosystems).

### SDS-PAGE and western blot analysis

Mosquito ovaries were dissected in 1× PBS under a dissecting microscope and homogenized in lysis buffer (12 mM sodium deoxycholate, 0.2% SDS, 1% triton X-100, complete mini EDTA-free protease inhibitor; Roche Applied Science, Indianapolis, IN, USA). Protein extracts were resolved on SDS-PAGE using a 12% acrylamide separation gel and a 3% stacking gel. The resolved proteins were either stained with GelCode Blue reagent (Thermo Fisher Scientific) or electrophoretically blotted to a nitrocellulose membrane (LI-COR, Lincoln, NE, USA) for western blot analysis. The membranes were blocked with 4% nonfat dry milk and incubated with each primary antibody in 4% nonfat milk in PBS containing 0.1% Tween 20. The EOF1 rabbit polyclonal antibody was generated by GenScript Corporation (Piscataway, NJ, USA) based on an antigenic peptide (LAPNSPSKEDEPAH). The anti-α-tubulin monoclonal antibody from Developmental Studies Hybridoma Bank (University of Iowa, Iowa City, IA, USA) was used as loading controls for ovaries. The dilutions of the primary antibodies were as follows: EOF1 (1:3,000) and α-tubulin (1:2,000). The secondary antibodies were either IRDye 800CW goat anti-rabbit secondary antibody (1:10,000; LI-COR) or IRDye 800CW goat anti-mouse secondary antibody (1:10,000; LI-COR). The protein bands were visualized with an Odyssey Infrared Imaging System (LI-COR).

### Measuring knockdown efficiency of RNAi-EOF1

Knockdown efficiency of RNAi was verified by real-time qPCR using gene-specific primers (S2 Table). cDNA was synthesized from DNase-I–treated total RNA isolated from ovaries of individual dsRNA-injected mosquitoes at 48 h PBM. Normalization was done using the ribosomal protein S7 transcript levels as an internal control, and the knockdown efficiency of RNAi-EOF1 was compared using Fluc-dsRNA–injected mosquitoes as a control. The RNAi knockdown level of EOF1 protein was also determined by western blot analysis using an EOF1-specific polyclonal antibody. Ovarian protein extracts were isolated from 8 individual mosquitoes from RNAi-Fluc or RNAi-EOF1 mosquitoes at 48 h PBM. α-tubulin was used as an internal control.

### Mosquito-egg–hatching assay

Eggs laid on oviposition papers remained wet for 3 days before drying at 28°C. Eggs (about 7 days old) on oviposition paper were submerged in water, vacuumed using a Speed Vac for 10 minutes, and allowed to hatch for 2 days. First-instar larvae were counted.

### Bleach assay

A bleach assay was performed to determine viability of 4-day-old *A*. *aegypti* eggs from RNAi studies. Eggs on oviposition paper were soaked in 12% bleach (sodium hypochlorite concentration at 0.72%) at room temperature. A gradual progress of dechorionation of eggshell was observed under a microscope. Light microscopic images of eggs deposited from RNAi-Fluc and RNAi-EOF1 females prior to and after the addition of bleach were taken at 49× magnification (Nikon, SMZ-10A; Nikon, Tokyo, Japan).

### FISH

mRNA distribution of EOF1 and vitelline envelopes (15a1, 15a2, and 15a3) in *A*. *aegypti* primary follicles was determined using whole-mount FISH. Primary follicles were isolated from ovaries of untreated female mosquitoes at 36 h PBM fixed with 4% paraformaldehyde. After washing with 1× PBS, the follicle samples were dehydrated with ethanol (ETOH) in water through a graded series for 10 min each in 10%, 30%, 50%, 70%, and 90% ETOH and 3 times 30 min each in 100% ETOH at room temperature. The samples were hydrated with 1× PBS in ETOH through a graded series for 20 min each in 25%, 50%, 75%, and 100% 1× PBS at room temperature. The follicles were permeabilized with proteinase K, postfixed with 4% paraformaldehyde with 0.1% Tween 20, and treated with DEPC (0.1%) to inactivate RNase. The follicles were then hybridized with digoxigenin-labeled antisense or sense RNA probes overnight at 65 ^o^C. Probe DNA templates were PCR amplified by gene-specific primers (S3 Table), and the RNA probes were synthesized by in vitro transcription as described in dsRNA synthesis above with DIG RNA Labeling Mix (Sigma-Aldrich, St. Louis, MO, USA). After washing, the follicles in PBS were stained for actin cytoskeleton using Acti-stain 488 phalloidin-labeled (Cytoskeleton, Denver, CO, USA) at room temperature and incubated with rhodamine-B–conjugated anti-digoxygenin antibody (1:500 dilution; Jackson ImmunoResearch Laboratories, West Grove, PA, USA) in blocking buffer (LI-COR) to detect the hybridized probes at 4 ^o^C. The follicles were mounted on glass slides and viewed on a spinning disc confocal microscope (Intelligent Imaging Innovations, Denver, CO, USA) at the Keck Imaging Center at the University of Arizona. Images were obtained by using excitation 488 and 561 nm lasers and recorded using identical exposure times (100 ms).

### Apoptosis assay for ovarian follicles using confocal laser microscopy

Female *A*. *aegypti* mosquitoes were microinjected with dsRNA at 1 day post-adult emergence, and ovaries at 36 h PBM were removed in 1× PBS under a dissecting microscope and immediately incubated with tissue culture media (Medium 199, Thermo Fisher Scientific) containing a caspase inhibitor (SR FLICA Poly Caspase Assay Kit; ImmunoChemistry Technologies, Bloomington, MN, USA) at 37 ^o^C in the dark for 1 h. The ovaries were washed with 1× PBS, fixed with 4% paraformaldehyde, quenched with 25 mM glycine, permeabilized with 0.5% Triton X100, and stained with Acti-stain 488 phalloidin overnight at 4 ^o^C. After washing with 1× PBS, the whole ovaries were mounted on a glass slide using ProLong Gold Antifade reagent (Thermo Fisher Scientific). Immunofluorescence, differential interference contrast, and light microscopic images of the ovaries were captured using a Spinning Disk Confocal Laser Microscope in the Keck Imaging Center at the University of Arizona.

### Rhodamine B and neutral red mosquito follicle permeability assay

The assay has an advantage in that it can quickly assess whether follicles within the ovaries may contain defective eggshell prior to oviposition. Individual follicles of untreated, RNAi-Fluc, or RNAi-EOF1 mosquitoes at 96 h PBM were dissected and gently separated from the ovaries and transferred to glass scintillation vials. Rhodamine B (final concentration of 1 mM in H_2_O, Sigma-Aldrich) and neutral red (0.5%, Sigma-Aldrich) were used to stain primary follicles for 10 min on a rocking shaker and thoroughly rinsed with H_2_O. The stained primary follicles were photographed with a Coolpix 4300 (Nikon).

### Ultrastructural study of eggshell by SEM

The ovaries were dissected from mosquitoes injected with Fluc control dsRNA and EOF1 dsRNA at 96 h PBM. Each follicle was carefully separated from the ovaries in 1× PBS under a dissecting microscope. The mature follicles were fixed in 2.5% glutaraldehyde in 0.1 M PIPES for 1 h at room temperature and washed twice in PIPES. The follicles were then postfixed in 1% osmium tetroxide in PIPES for 1 h and washed twice in deionized water for 10 min each. The follicles were dehydrated with ETOH in water through a graded series for 10 min each in 10%, 30%, 50%, 70%, and 90% ETOH and 3 times 30 min each in 100% ETOH at room temperature. The samples were dried with hexamethyldisilazane (HMDS; Electron Microscopy Sciences, Hatfield, PA, USA) in ETOH through a graded series for 20 min each in 25%, 50%, 75%, and 100% HMDS at room temperature. The follicle samples were air dried under a fume hood overnight at room temperature for SEM analysis. The dried samples were metallized with gold using Hummer 6.2 Sputter System (Anatech USA, Union City, CA, USA). Inspect-S scanning electron microscope (FEI, Hillsboro, OR, USA) was used to compare the ultrastructural characteristics of the ovarian follicles of females injected with Fluc and EOF1 dsRNA.

### Preparation of enriched mosquito eggshell

Female mosquitoes were injected with dsRNA at 1 day post-adult emergence, and ovaries were dissected in 1× PBS at 96 h PBM. The dissected ovaries were thoroughly homogenized (40 strokes) in ice-cold 1× PBS using Dounce homogenizers (B pestle). The eggshells were allowed to settle down to the bottom of the homogenizer on ice. The top cloudy fraction was gently aspirated, and the washing step with ice-cold 1× PBS for the eggshells was repeated four times or until the solution was completely cleared. Subsequently, the eggshell was homogenized (20 strokes) in ice-cold 1× PBS using A pestle. The eggshell proteins were subjected to SDS-PAGE and stained with GelCode Blue reagent.

### Statistical analysis

Statistical analyses were performed using GraphPad Prism Software (GraphPad, La Jolla, CA). Statistical significance for fecundity, viability, and RNAi knockdown efficiency was analyzed using an unpaired Student's *t* test. *p* values of ≤0.05 were considered significantly different. All experiments were performed from at least three independent cohorts.

## Supporting information

S1 Fig A confocal microscopic image of an ovariole isolated from *A*. *aegypti* mosquito. The representative image shows a developing primary follicle, a resting secondary follicle, and a germarium at 36 h PBM. Fixed follicles were stained for actin (phalloidin, green) and cell nuclei (DAPI, purple), and images were obtained by Nikon C1si confocal laser scanning microscopy at the Keck Imaging Center at the University of Arizona (scale bar: 100 μm). PBM, post-blood meal.
(TIF)

S2 Fig EOF1 protein is necessary for complete eggshell formation and embryonic development in *A*. *aegypti* mosquitoes. A bleach test was performed to determine viability of 4-day-old eggs from RNAi studies. Light microscopic images were taken from RNAi-Fluc and RNAi-EOF1 females immediately prior to the addition of bleach (0 min). We frequently observed that some partially melanized eggs from EOF1 deficient mosquitoes collapsed prior to bleach application. (A) Representative photos were taken 2, 50, 60, 70, and 80 min post-bleach application. The exochorionic structures, including EN, become invisible immediately upon bleach application (2 min). Eyes of the first-instar larvae present in eggs, indicated with white circles, have begun to appear through the partially dechorionated eggshell at 50 min post-bleach application, while weakly melanized eggs from EOF1-deficient mosquitoes disappeared. The eggshell was nearly removed by 80 min after bleach treatment, exposing the fully developed first-instar larvae. Bleach treatment (10%) gently dechorionates eggshell with minimal adverse effects on the embryos due to the presence of the extraembryonic serosal cuticle. (B) Presence of larvae was determined. Overall, the bleach studies showed that eggs from RNAi-Fluc mosquitoes had 92.2% of developed first-instar larvae, while 1.8% of egg deposited by RNAi-EOF1 mosquitoes successfully completed embryogenesis to reach the first larval instar. Ten egg papers from both groups were treated with bleach. The mean ± SE are shown as horizontal lines, and the statistical significance is represented by stars above each column (unpaired Student's *t* test; ***p < 0.001). Eggs were observed using a light microscope at 49× magnification (Nikon, SMZ-10A). Underlying data can be found in S1 Data. EN, exochorionic network; EOF1, eggshell organizing factor 1; Fluc, firefly luciferase; RNAi, RNA interference; SE, standard error.
(TIF)

S3 Fig Reproductive phenotypes associated with EOF1 gene silencing by RNAi in *A*. *aegypti* mosquitoes. (A) Mosquitoes injected with dsRNA-EOF1 at one day after adult eclosion produced inviable eggs. (B) Mosquitoes were injected with dsRNA-EOF1 immediately after blood feeding. These females laid eggs that show no difference in fecundity and viability compared to RNAi-Fluc control mosquitoes. (C) Mosquitoes injected with dsRNA-EOF1 at 48 h PBM and before oviposition laid normal eggs. (D) Mosquitoes injected with dsRNA-EOF1 at 1 day after oviposition resulted in the production of inviable eggs. The schematic images show an oviposition experimental setup. Representative eggs are shown from each dsRNA injection experiment. The effect of RNAi-Fluc control or RNAi-EOF1 on *A*. *aegypti* fecundity was examined by counting the number of eggs laid by each individual female. Each dot represents the number of eggs oviposited by an individual mosquito (*N* = 30). Viability of these eggs was determined. Each bar corresponds to egg viability from 15 individual mosquitoes from two groups. The mean ± SE are shown as horizontal lines. Statistical significance is represented by stars above each column (unpaired Student's *t* test; ***p < 0.001). Underlying data can be found in S1 Data. dsRNA, double-stranded RNA; EOF1, eggshell organizing factor 1; Fluc, firefly luciferase; NS, not significant; PBM, post-blood meal; RNAi, RNA interference; SE, standard error.
(TIF)

S4 Fig Programmed shedding of follicular epithelial cells, secondary follicles, and germarium from the primary follicle occurs during late oocyte development in *A*. *aegypti*. Confocal microscopy images of representative individual follicles during mid- to late oogenesis, showing follicle development and shedding of the follicular epithelial cell layer. Ovaries were dissected from untreated wild-type females between 24 and 72 h PBM and carefully teased to obtain individual follicles in 1× PBS. The follicles were immediately fixed with 4.0% paraformaldehyde and stained for actin cytoskeleton (phalloidin, excitation wavelength of 488 nm, green) and cell nuclei (DAPI, excitation wavelength of 403 nm, purple). Images were obtained by Nikon C1si confocal laser scanning microscopy at the Keck Imaging Center at the University of Arizona. Scale bar corresponds to 100 μm. PBM, post-blood meal.
(TIF)

S5 Fig mRNA distribution of EOF1 in *A*. *aegypti* mosquito primary follicles using whole-mount FISH. (A) EOF1 mRNA transcript distributions in primary follicles were visualized by hybridizing digoxygenin-labeled RNA probes. Primary follicles isolated from ovaries of untreated female mosquitoes at 36 h PBM were fixed with 4% paraformaldehyde and hybridized with digoxigenin-labeled antisense or sense RNA probes. The follicles were stained for actin cytoskeleton using Acti-stain 488 phalloidin-labeled (Cytoskeleton) and incubated with rhodamine-B–conjugated anti-digoxygenin antibody (Jackson ImmunoResearch Laboratories) to detect the hybridized probes. The mRNA distributions of 15a1 (B), 15a2 (C), and 15a3 (D) vitelline envelope proteins were also determined in fixed follicles. The DIC (above) and merged fluorescent images (below) illustrate that EOF1 mRNA transcripts are present in oocyte and nurse cells of primary follicles and weakly expressed in the secondary follicle, while mRNAs encoding three vitelline envelope proteins are restricted in follicular epithelial cells of primary follicles. Follicles were viewed on a spinning disc confocal microscope (Intelligent Imaging Innovations) at the Keck Imaging Center at the University of Arizona. Images were obtained by using excitation with 488 and 561 nm lasers and recorded using identical exposure times (100 ms). Scale bars = 50 μm. DIC, differential interference contrast; EOF1, eggshell organizing factor 1; FISH, fluorescent in situ hybridization; mRNA, messenger RNA; PBM, post-blood meal.
(TIF)

S6 Fig RNAi-mediated knockdown of EOF1 expression in *A*. *aegypti* ovaries. (A) Single-mosquito qPCR analysis was performed to measure the relative RNAi knockdown level of EOF1 transcript in ovaries. Mosquitoes were microinjected with 2.0 μg of dsRNA-Fluc or dsRNA-EOF1 three days prior to blood feeding, and a pair of ovaries was dissected from 13 individual mosquitoes from both groups at 48 h PBM. EOF1 transcript levels were normalized to S7 ribosomal protein transcript levels in the same cDNA samples. The mean ± SE are shown as horizontal lines. Statistical significance is represented by asterisks above the column (unpaired Student's *t* test; ***p < 0.001). (B) Western blot analysis was performed to determine the RNAi knockdown level of EOF1 protein in ovaries using an EOF1-specific polyclonal antibody. Ovarian protein extracts from 8 mosquitoes treated either with dsRNA-Fluc or dsRNA-EOF1 at 48 h PBM were loaded and analyzed individually by SDS-PAGE. Each lane contains 0.3 ovary equivalent of protein extracts. α-tubulin was used as an internal control. Underlying data can be found in S1 Data. cDNA, complementary DNA; EOF1, eggshell organizing factor 1; Fluc, firefly luciferase; PBM, post-blood meal; qPCR, quantitative real-time PCR; RNAi, RNA interference; SE, standard error.
(TIF)

S7 Fig Reproductive phenotypes associated with EOF1 gene silencing by RNAi in *A*. *albopictus*. (A) Representative eggs are shown from mosquitoes injected with dsRNA-Fluc and dsRNA-EOF1. Mosquitoes were injected with dsRNA at 1 day after adult eclosion. (B) The effect of RNAi-Fluc control or RNAi-EOF1 control on *A*. *albopictus* fecundity was examined by counting the number of eggs laid by each individual female. Each dot represents the number of eggs oviposited by an individual mosquito (*N* = 15). The mean ± SE are shown as horizontal lines. Statistical significance is represented by stars above each column (unpaired Student's *t* test; ***p < 0.001). RNAi knockdown of EOF1 in *A*. *albopictus* females led to the production of nonmelanized abnormal eggs. Note that 55.9% of fully blood-fed RNAi-EOF1 females did not produce mature follicles, and the results are not included in the analysis. (C) Viability of these eggs was determined. Each bar corresponds to egg viability from 10 individual mosquitoes from both groups. (D) *A*. *albopictus* (Gainesville strain, MRA-804) obtained from the CDC is presented, showing a distinct single longitudinal white stripe on the dorsal thorax. Underlying data can be found in S1 Data. dsRNA, double-stranded RNA; EOF1, eggshell organizing factor 1; Fluc, firefly luciferase; RNAi, RNA interference; SE, standard error.
(TIF)

S8 Fig Eggshell enrichment and SDS-PAGE protein analysis. Representative ovaries from mosquitoes injected with dsRNA-Fluc (A) and dsRNA-EOF1 (A′). Mosquitoes were injected with dsRNA targeting EOF1 or Fluc control, and the ovaries were dissected from the injected mosquitoes at 96 h PBM. Ovaries were photographed immediately after dissection in 1× PBS. As shown in Fig 5, RNAi-Fluc control mosquitoes contain mature nonmelanized ovarian follicles, whereas EOF1-deficient mosquitoes contain aberrant prematurely melanized follicles. (B and B′) Images show individual follicles that were removed from ovaries prior to eggshell enrichment. (C and C′) Enrichment of mosquito eggshell was achieved by homogenizing follicles with a Dounce homogenizer (B pestle) and washing cytosolic contents. (D) SDS-PAGE analysis of enriched eggshell proteins. Proteins equivalent to two ovaries were loaded in each well. Red arrows indicate possible eggshell proteins that are affected in response to RNAi-EOF1 compared to RNAi-FLUC control. dsRNA, double-stranded RNA; EOF1, eggshell organizing factor 1; Fluc, firefly luciferase; PBM, post-blood meal; RNAi, RNA interference.
(TIF)

S1 Table Gene-specific primers used to amplify DNA templates for dsRNA synthesis. dsRNA, double-stranded RNA.
(XLSX)

S2 Table Gene-specific primers for RNAi and qPCR. qPCR, quantitative real-time PCR; RNAi, RNA interference.
(XLSX)

S3 Table Gene-specific primers used to synthesize in situ hybridization probes. (XLSX)

S1 Data Numerical data used in the figures. (XLSX)

## Abbreviations

cDNA: complementary DNA
CRISPR-Cas9: clustered regularly interspaced short palindromic repeats and CRISPR-associated protein 9
CT: central tubercle
DIC: differential interference contrast
dsRNA: double-stranded RNA
EN: exochorionic network
EOF1: eggshell organizing factor 1
EST: expressed sequence tag
ETOH: ethanol
FISH: fluorescent in situ hybridization
Fluc: firefly luciferase
HMDS: hexamethyldisilazane
mRNA: messenger RNA
OCA: outer chorionic area
PBM: post-blood meal
PT: peripheral tubercle
qPCR: quantitative real-time PCR
RNAi: RNA interference
SCF complex: Skp, Cullin, F-box containing complex
SE: standard error
SEM: scanning electron microscopy
TSA: transcriptome shotgun assembly
