# Supplementary figures and images for: Identification and characterization of a mosquito-specific eggshell organizing factor in Aedes aegypti mosquitoes
Source: PLoS Biol. 2019 Jan 8;17(1):e3000068. doi: 10.1371/journal.pbio.3000068 (PMC6324781; doi:10.1371/journal.pbio.3000068)

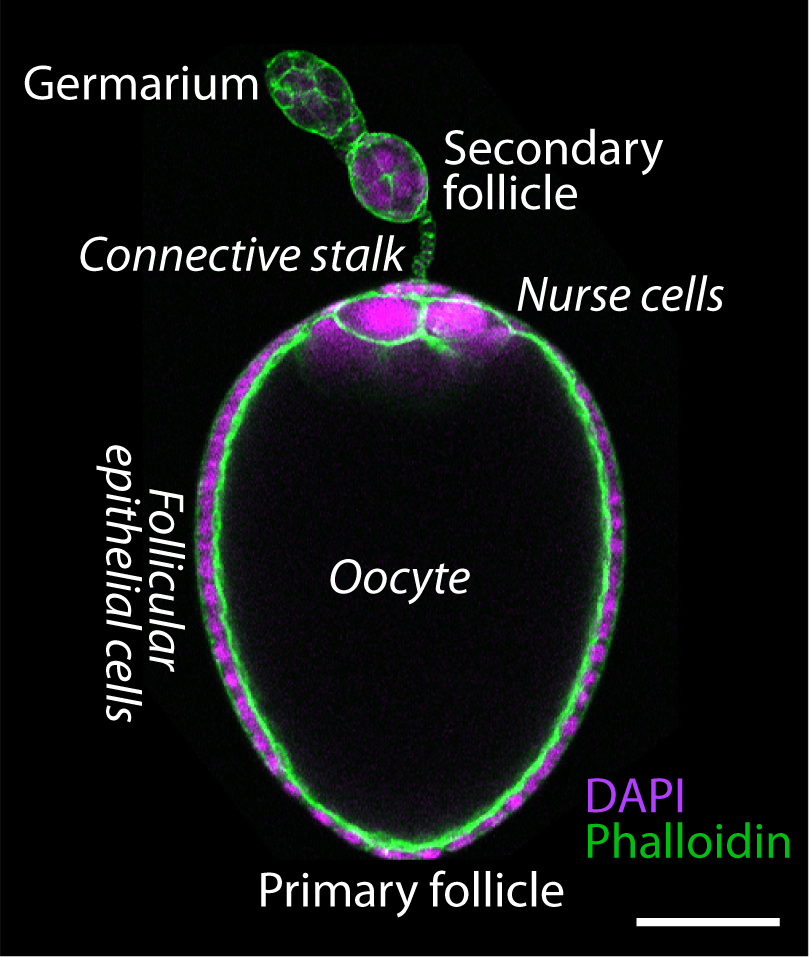

Supplement: S1 Fig — The representative image shows a developing primary follicle, a resting secondary follicle, and a germarium at 36 h PBM. Fixed follicles were stained for actin (phalloidin, green) and cell nuclei (DAPI, purple), and images were obtained by Nikon C1si confocal laser scanning microscopy at the Keck Imaging Center at the University of Arizona (scale bar: 100 μm). PBM, post-blood meal. (TIF) [file pbio.3000068.s001.tif]

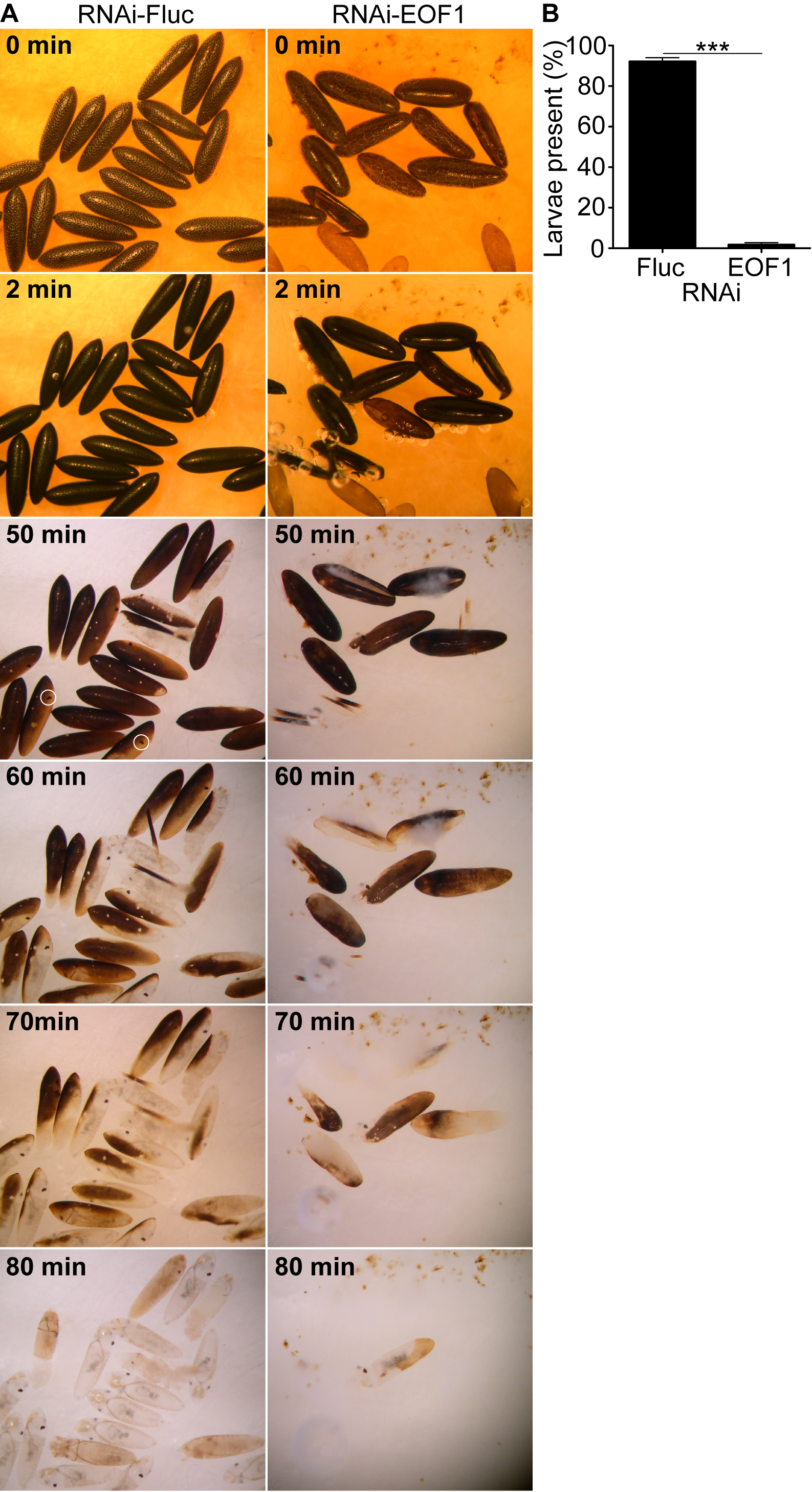

Supplement: S2 Fig — A bleach test was performed to determine viability of 4-day-old eggs from RNAi studies. Light microscopic images were taken from RNAi-Fluc and RNAi-EOF1 females immediately prior to the addition of bleach (0 min). We frequently observed that some partially melanized eggs from EOF1 deficient mosquitoes collapsed prior to bleach application. (A) Representative photos were taken 2, 50, 60, 70, and 80 min post-bleach application. The exochorionic structures, including EN, become invisible immediately upon bleach application (2 min). Eyes of the first-instar larvae present in eggs, indicated with white circles, have begun to appear through the partially dechorionated eggshell at 50 min post-bleach application, while weakly melanized eggs from EOF1-deficient mosquitoes disappeared. The eggshell was nearly removed by 80 min after bleach treatment, exposing the fully developed first-instar larvae. Bleach treatment (10%) gently dechorionates eggshell with minimal adverse effects on the embryos due to the presence of the extraembryonic serosal cuticle. (B) Presence of larvae was determined. Overall, the bleach studies showed that eggs from RNAi-Fluc mosquitoes had 92.2% of developed first-instar larvae, while 1.8% of egg deposited by RNAi-EOF1 mosquitoes successfully completed embryogenesis to reach the first larval instar. Ten egg papers from both groups were treated with bleach. The mean ± SE are shown as horizontal lines, and the statistical significance is represented by stars above each column (unpaired Student's t test; ***p < 0.001). Eggs were observed using a light microscope at 49× magnification (Nikon, SMZ-10A). Underlying data can be found in S1 Data. EN, exochorionic network; EOF1, eggshell organizing factor 1; Fluc, firefly luciferase; RNAi, RNA interference; SE, standard error. (TIF) [file pbio.3000068.s002.tif]

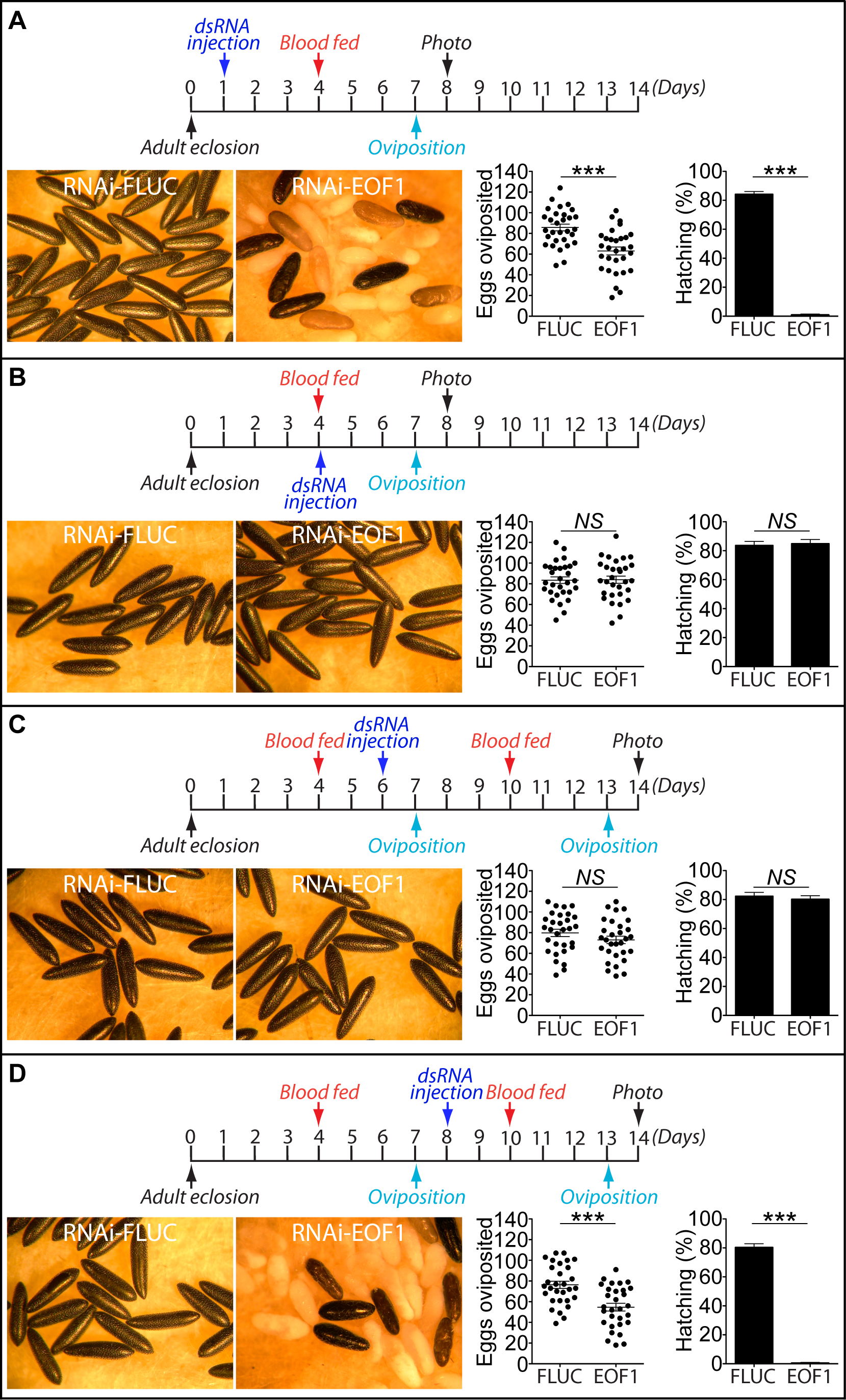

Supplement: S3 Fig — (A) Mosquitoes injected with dsRNA-EOF1 at one day after adult eclosion produced inviable eggs. (B) Mosquitoes were injected with dsRNA-EOF1 immediately after blood feeding. These females laid eggs that show no difference in fecundity and viability compared to RNAi-Fluc control mosquitoes. (C) Mosquitoes injected with dsRNA-EOF1 at 48 h PBM and before oviposition laid normal eggs. (D) Mosquitoes injected with dsRNA-EOF1 at 1 day after oviposition resulted in the production of inviable eggs. The schematic images show an oviposition experimental setup. Representative eggs are shown from each dsRNA injection experiment. The effect of RNAi-Fluc control or RNAi-EOF1 on A. aegypti fecundity was examined by counting the number of eggs laid by each individual female. Each dot represents the number of eggs oviposited by an individual mosquito (N = 30). Viability of these eggs was determined. Each bar corresponds to egg viability from 15 individual mosquitoes from two groups. The mean ± SE are shown as horizontal lines. Statistical significance is represented by stars above each column (unpaired Student's t test; ***p < 0.001). Underlying data can be found in S1 Data. dsRNA, double-stranded RNA; EOF1, eggshell organizing factor 1; Fluc, firefly luciferase; NS, not significant; PBM, post-blood meal; RNAi, RNA interference; SE, standard error. (TIF) [file pbio.3000068.s003.tif]

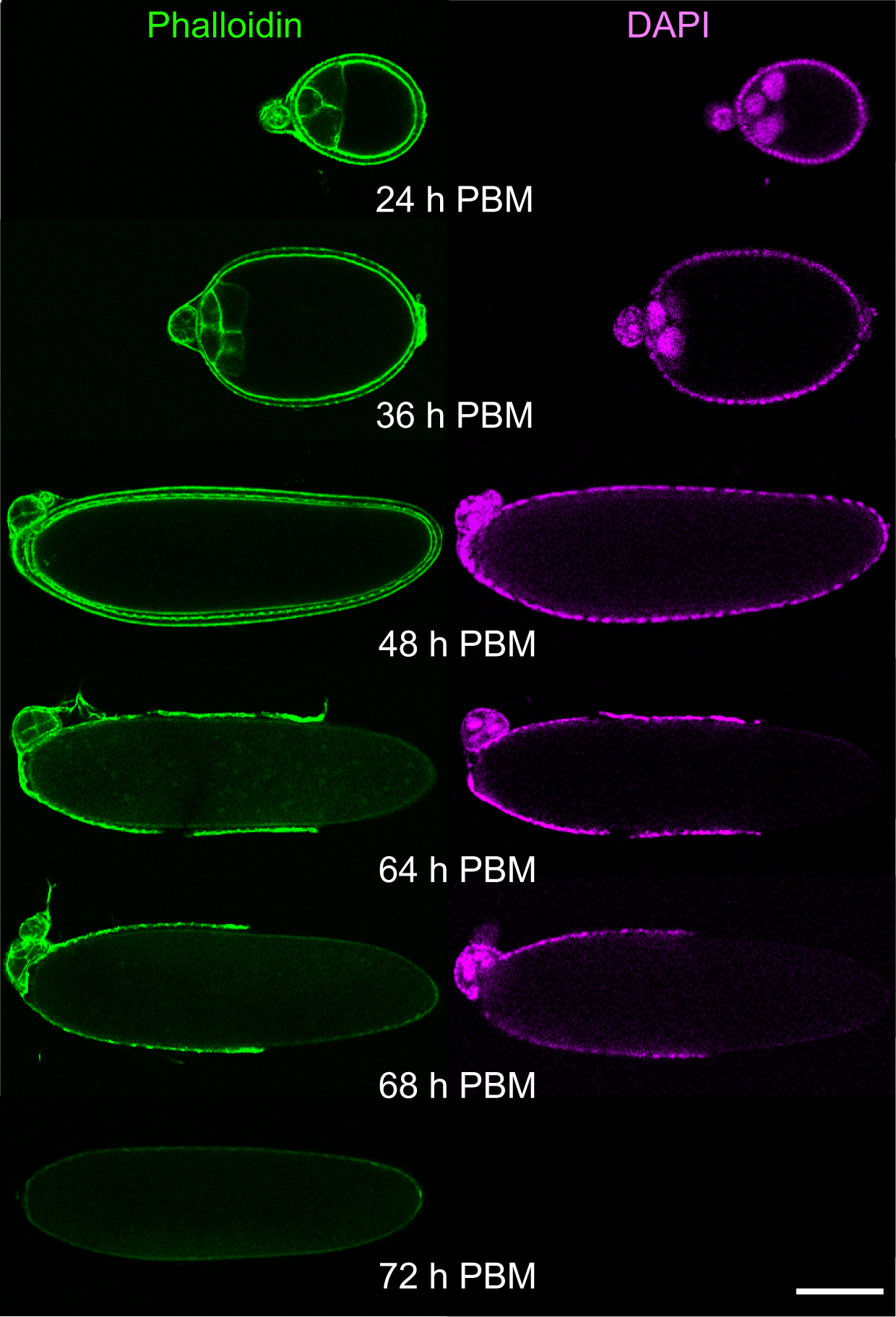

Supplement: S4 Fig — Confocal microscopy images of representative individual follicles during mid- to late oogenesis, showing follicle development and shedding of the follicular epithelial cell layer. Ovaries were dissected from untreated wild-type females between 24 and 72 h PBM and carefully teased to obtain individual follicles in 1× PBS. The follicles were immediately fixed with 4.0% paraformaldehyde and stained for actin cytoskeleton (phalloidin, excitation wavelength of 488 nm, green) and cell nuclei (DAPI, excitation wavelength of 403 nm, purple). Images were obtained by Nikon C1si confocal laser scanning microscopy at the Keck Imaging Center at the University of Arizona. Scale bar corresponds to 100 μm. PBM, post-blood meal. (TIF) [file pbio.3000068.s004.tif]

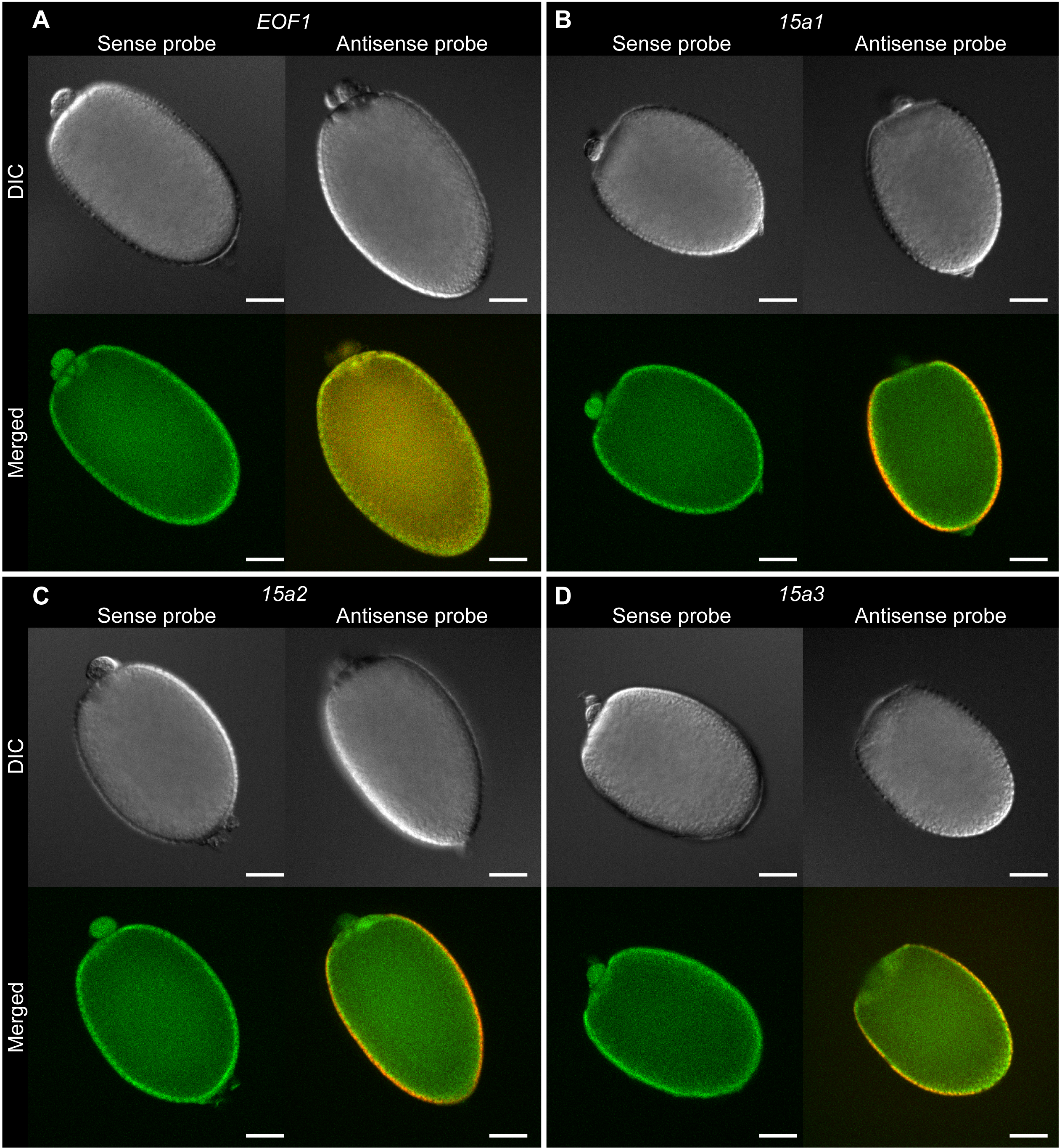

Supplement: S5 Fig — (A) EOF1 mRNA transcript distributions in primary follicles were visualized by hybridizing digoxygenin-labeled RNA probes. Primary follicles isolated from ovaries of untreated female mosquitoes at 36 h PBM were fixed with 4% paraformaldehyde and hybridized with digoxigenin-labeled antisense or sense RNA probes. The follicles were stained for actin cytoskeleton using Acti-stain 488 phalloidin-labeled (Cytoskeleton) and incubated with rhodamine-B–conjugated anti-digoxygenin antibody (Jackson ImmunoResearch Laboratories) to detect the hybridized probes. The mRNA distributions of 15a1 (B), 15a2 (C), and 15a3 (D) vitelline envelope proteins were also determined in fixed follicles. The DIC (above) and merged fluorescent images (below) illustrate that EOF1 mRNA transcripts are present in oocyte and nurse cells of primary follicles and weakly expressed in the secondary follicle, while mRNAs encoding three vitelline envelope proteins are restricted in follicular epithelial cells of primary follicles. Follicles were viewed on a spinning disc confocal microscope (Intelligent Imaging Innovations) at the Keck Imaging Center at the University of Arizona. Images were obtained by using excitation with 488 and 561 nm lasers and recorded using identical exposure times (100 ms). Scale bars = 50 μm. DIC, differential interference contrast; EOF1, eggshell organizing factor 1; FISH, fluorescent in situ hybridization; mRNA, messenger RNA; PBM, post-blood meal. (TIF) [file pbio.3000068.s005.tif]

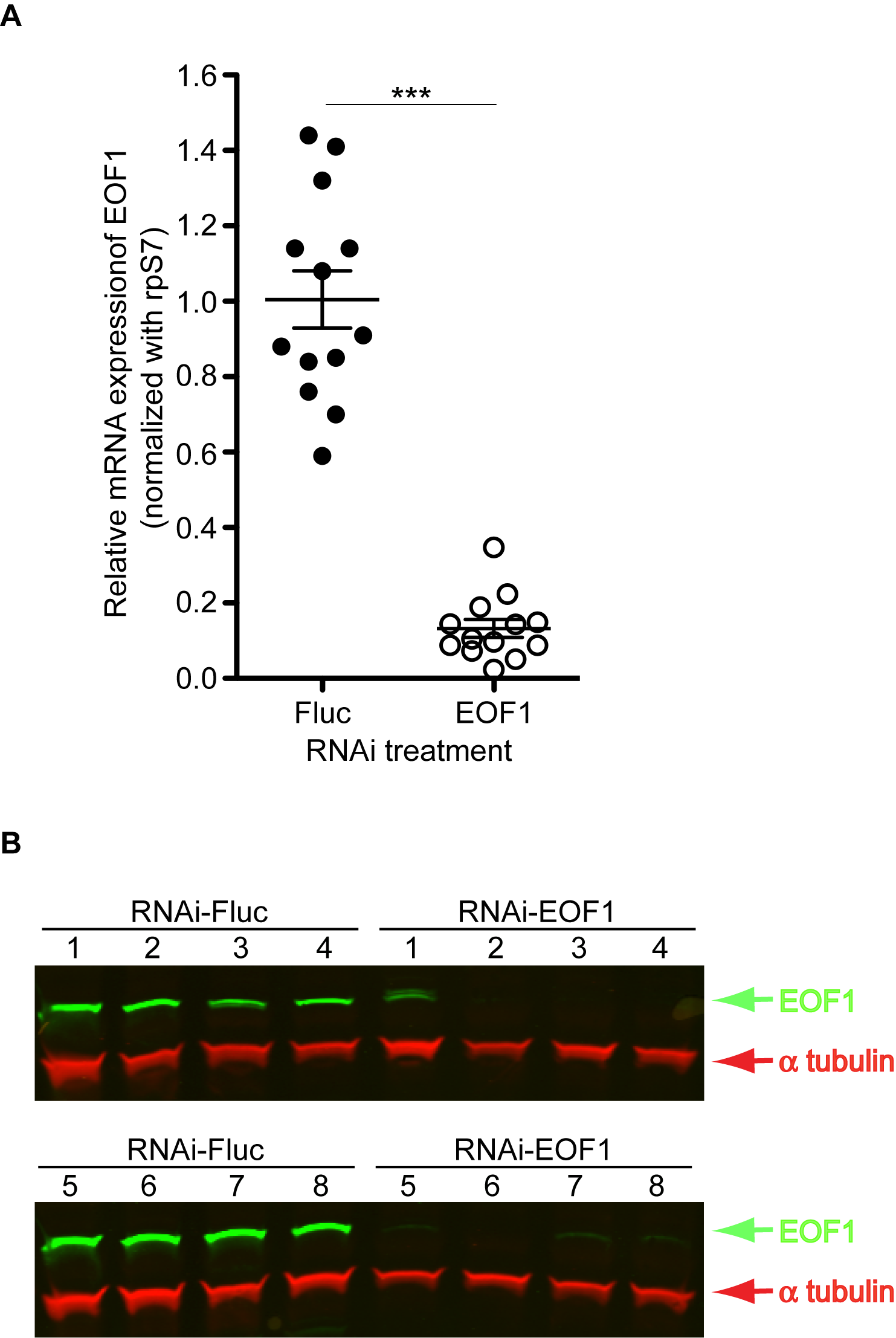

Supplement: S6 Fig — (A) Single-mosquito qPCR analysis was performed to measure the relative RNAi knockdown level of EOF1 transcript in ovaries. Mosquitoes were microinjected with 2.0 μg of dsRNA-Fluc or dsRNA-EOF1 three days prior to blood feeding, and a pair of ovaries was dissected from 13 individual mosquitoes from both groups at 48 h PBM. EOF1 transcript levels were normalized to S7 ribosomal protein transcript levels in the same cDNA samples. The mean ± SE are shown as horizontal lines. Statistical significance is represented by asterisks above the column (unpaired Student's t test; ***p < 0.001). (B) Western blot analysis was performed to determine the RNAi knockdown level of EOF1 protein in ovaries using an EOF1-specific polyclonal antibody. Ovarian protein extracts from 8 mosquitoes treated either with dsRNA-Fluc or dsRNA-EOF1 at 48 h PBM were loaded and analyzed individually by SDS-PAGE. Each lane contains 0.3 ovary equivalent of protein extracts. α-tubulin was used as an internal control. Underlying data can be found in S1 Data. cDNA, complementary DNA; EOF1, eggshell organizing factor 1; Fluc, firefly luciferase; PBM, post-blood meal; qPCR, quantitative real-time PCR; RNAi, RNA interference; SE, standard error. (TIF) [file pbio.3000068.s006.tif]

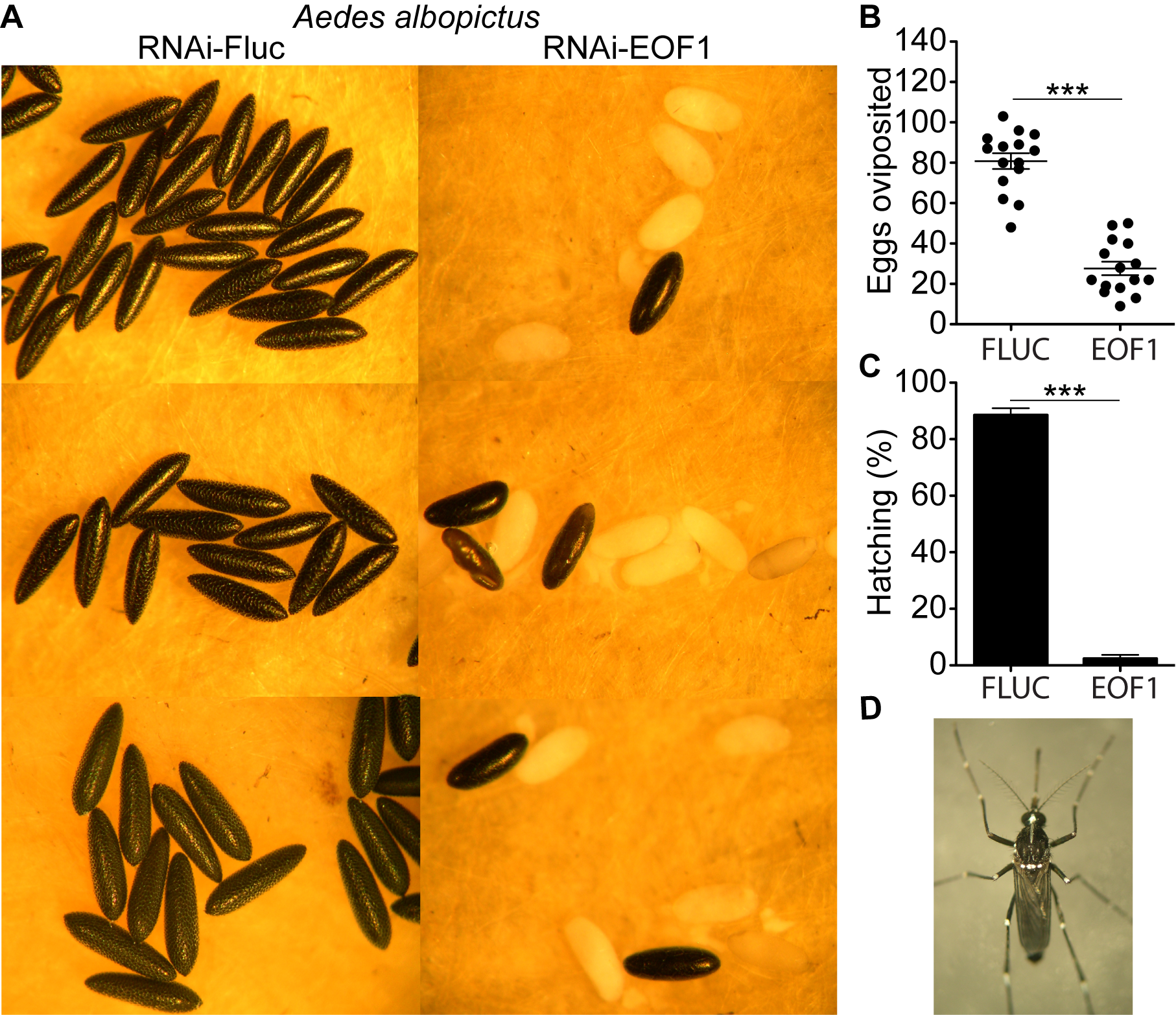

Supplement: S7 Fig — (A) Representative eggs are shown from mosquitoes injected with dsRNA-Fluc and dsRNA-EOF1. Mosquitoes were injected with dsRNA at 1 day after adult eclosion. (B) The effect of RNAi-Fluc control or RNAi-EOF1 control on A. albopictus fecundity was examined by counting the number of eggs laid by each individual female. Each dot represents the number of eggs oviposited by an individual mosquito (N = 15). The mean ± SE are shown as horizontal lines. Statistical significance is represented by stars above each column (unpaired Student's t test; ***p < 0.001). RNAi knockdown of EOF1 in A. albopictus females led to the production of nonmelanized abnormal eggs. Note that 55.9% of fully blood-fed RNAi-EOF1 females did not produce mature follicles, and the results are not included in the analysis. (C) Viability of these eggs was determined. Each bar corresponds to egg viability from 10 individual mosquitoes from both groups. (D) A. albopictus (Gainesville strain, MRA-804) obtained from the CDC is presented, showing a distinct single longitudinal white stripe on the dorsal thorax. Underlying data can be found in S1 Data. dsRNA, double-stranded RNA; EOF1, eggshell organizing factor 1; Fluc, firefly luciferase; RNAi, RNA interference; SE, standard error. (TIF) [file pbio.3000068.s007.tif]

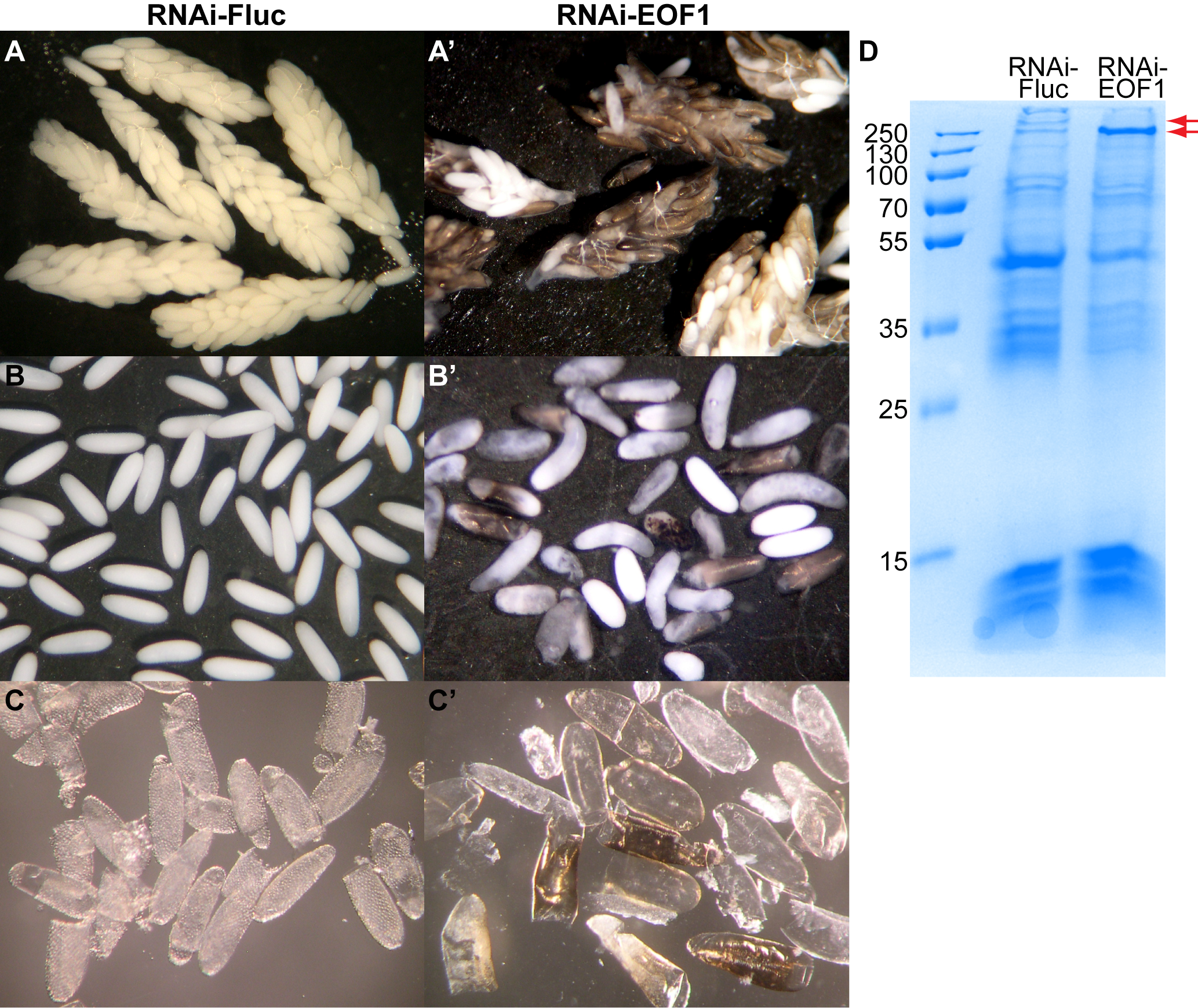

Supplement: S8 Fig — Representative ovaries from mosquitoes injected with dsRNA-Fluc (A) and dsRNA-EOF1 (A′). Mosquitoes were injected with dsRNA targeting EOF1 or Fluc control, and the ovaries were dissected from the injected mosquitoes at 96 h PBM. Ovaries were photographed immediately after dissection in 1× PBS. As shown in Fig 5, RNAi-Fluc control mosquitoes contain mature nonmelanized ovarian follicles, whereas EOF1-deficient mosquitoes contain aberrant prematurely melanized follicles. (B and B′) Images show individual follicles that were removed from ovaries prior to eggshell enrichment. (C and C′) Enrichment of mosquito eggshell was achieved by homogenizing follicles with a Dounce homogenizer (B pestle) and washing cytosolic contents. (D) SDS-PAGE analysis of enriched eggshell proteins. Proteins equivalent to two ovaries were loaded in each well. Red arrows indicate possible eggshell proteins that are affected in response to RNAi-EOF1 compared to RNAi-FLUC control. dsRNA, double-stranded RNA; EOF1, eggshell organizing factor 1; Fluc, firefly luciferase; PBM, post-blood meal; RNAi, RNA interference. (TIF) [file pbio.3000068.s008.tif]
